# Cecropin D-derived synthetic peptides in the fight against *Candida albicans* cell filamentation and biofilm formation

**DOI:** 10.3389/fmicb.2022.1045984

**Published:** 2023-01-13

**Authors:** Ibeth Guevara-Lora, Grazyna Bras, Magdalena Juszczak, Justyna Karkowska-Kuleta, Andrzej Gorecki, Marcela Manrique-Moreno, Jakub Dymek, Elzbieta Pyza, Andrzej Kozik, Maria Rapala-Kozik

**Affiliations:** ^1^Department of Analytical Biochemistry, Faculty of Biochemistry, Biophysics and Biotechnology, Jagiellonian University, Krakow, Poland; ^2^Department of Comparative Biochemistry and Bioanalytics, Faculty of Biochemistry, Biophysics and Biotechnology, Jagiellonian University, Krakow, Poland; ^3^Department of Physical Biochemistry, Faculty of Biochemistry, Biophysics and Biotechnology, Jagiellonian University, Krakow, Poland; ^4^Chemistry Institute, Faculty of Exact and Natural Sciences, University of Antioquia, Medellin, Colombia; ^5^Department of Cell Biology and Imaging, Institute of Zoology and Biomedical Research, Jagiellonian University, Krakow, Poland

**Keywords:** antimicrobial peptides, cecropin, *Candida*, candidiasis, biofilm

## Abstract

The recent progressive increase in the incidence of invasive fungal infections, especially in immunocompromised patients, makes the search for new therapies crucial in the face of the growing drug resistance of prevalent nosocomial yeast strains. The latest research focuses on the active compounds of natural origin, inhibiting fungal growth, and preventing the formation of fungal biofilms. Antimicrobial peptides are currently the subject of numerous studies concerning effective antifungal therapy. In the present study, the antifungal properties of two synthetic peptides (ΔM3, ΔM4) derived from an insect antimicrobial peptide – cecropin D – were investigated. The fungicidal activity of both compounds was demonstrated against the yeast forms of *Candida albicans*, *Candida tropicalis*, and *Candida parapsilosis*, reaching a MFC_99.9_ in the micromolar range, while *Candida glabrata* showed greater resistance to these peptides. The scanning electron microscopy revealed a destabilization of the yeast cell walls upon treatment with both peptides; however, their effectiveness was strongly modified by the presence of salt or plasma in the yeast environment. The transition of *C. albicans* cells from yeast to filamentous form, as well as the formation of biofilms, was effectively reduced by ΔM4. Mature biofilm viability was inhibited by a higher concentration of this peptide and was accompanied by increased ROS production, activation of the *GPX3* and *SOD5* genes, and finally, increased membrane permeability. Furthermore, both peptides showed a synergistic effect with caspofungin in inhibiting the metabolic activity of *C. albicans* cells, and an additive effect was also observed for the mixtures of peptides with amphotericin B. The results indicate the possible potential of the tested peptides in the prevention and treatment of candidiasis.

## 1. Introduction

The development of infections caused by *Candida* species consistently represents a serious threat, especially in individuals with different immunodeficiencies. Candidiases are generally treated with drugs such as azoles, polyenes, pyrimidine analogs, and echinocandins (Lee et al., 2021). However, when applied to *Candida* clinical isolates, the effectiveness of these drugs decreases significantly. The constant emergence of fungal strains resistant to classical treatment has prompted the search for new drugs for *Candida* infections, particularly among products of natural origin, including those from plants, insects, algae, etc. (Aldholmi et al., 2019; Guevara-Lora et al., 2020). These active substances include also antimicrobial peptides (AMPs), many of which have also shown antifungal activity (Bondaryk et al., 2017; Huan et al., 2020). A group of peptides that currently attracts particular attention for their potential role in the fight against candidiasis is cecropins (Brady et al., 2019). These peptides, identified currently in various insects, were originally isolated from the hemolymph of the *Hyalophora cecropia* moth (Boman et al., 1991) and are actually classified into several subfamilies – cecropins A, B, C, D, E and cecropin-like peptides (Romoli et al., 2017; Peng et al., 2019). These α-helical AMPs generally possess a strongly basic N-terminal region linked to a more hydrophobic C-terminal region through a segment rich in proline and glycine residues and their antimicrobial properties are attributed to their specific chemical structure (Boman et al., 1991; Sato and Feix, 2006). The presence of aromatic residues such as tryptophan and phenylalanine in the amphipathic N-terminal helix was shown to be essential for peptide-pathogen interactions, with subsequent permeabilization of the inner membrane of Gram-negative bacteria (Lee et al., 2015). The hydrophobic C-terminal region synergistically enhances this effect through the interaction with hydrophobic cell membrane. Although cecropins and cecropin-like peptides are more efficient in fighting Gram-negative microorganisms, the antimicrobial effects have also been shown for a broad range of Gram-positive bacteria (for a review, see Brady et al., 2019). Their activity against yeasts and other fungi was also proven, but the effects observed were not satisfactory. Most of the peptides demonstrating anticandidal activity belonged to the cecropin A or B subfamilies and required high concentration doses for effective action. Nevertheless, it was also shown that the use of analogs of these peptides could improve their antifungal properties (De Lucca et al., 2000; Ji et al., 2014), as certain modifications of the peptides, including the substitution of selected amino acid residues with tryptophan or D-amino acids, resulted in improved fungicidal activity due to the increased resistance to microbial proteolysis.

Promising antimicrobial properties were also previously reported for the cecropin D-like peptide (UniProtKB accession number: P85210) from the greater wax moth *Galleria mellonella* (Cytrynska et al., 2007). Modification of particular residues resulting in an increase of peptide charge and basic residues on the polar face of the molecule compared to a neutral wild-type peptide, enhanced the antibacterial activity of peptides derived from the cecropin D-like natural sequence. One such type of derivative was the synthetic cationic peptide ΔM2 with 39 amino acid residues and a charge of +9, which acts most effectively against Gram-negative bacteria (Oñate-Garzón et al., 2017). The necessity to continue the search for derivatives with enhanced antimicrobial properties, extended also to other bacterial and fungal pathogens, has led to further modifications. In this study, the anticandidal potential of two synthetic peptides specified as ΔM3 (NFFKRIRRAGKRIRKAIISA) and ΔM4 (NFFKRIRRAWKRIWKWIYSA), composed of 20 amino acids and derived from the N-terminal fragments of ΔM2, was analyzed. The ΔM3 peptide has a charge of +8 at physiological pH and was previously demonstrated to possess a high antimicrobial activity against *Staphylococcus aureus* and low hemolytic activity in human erythrocytes (Manrique-Moreno et al., 2021). The ΔM4 peptide with additional substitution of selected amino acids by three tryptophan and one tyrosine residues and a charge of +7 at physiological pH, presented anticancer properties against human melanoma cells (Santa-González et al., 2020). The helical-wheel projection of ΔM3 and ΔM4 and the predicted α-helical structure of the peptides generated with I-TASSER V5.1 software were presented in previous reports (Santa-González et al., 2020; Manrique-Moreno et al., 2021).

The main aim of this study was first to evaluate the fungicidal activity of ΔM3 and ΔM4 peptides against *Candida* species determining the minimal fungicidal concentration (MFC). Subsequently, the effects of different concentrations of peptides on *Candida albicans* hyphae, biofilm formation, and mature biofilm eradication were investigated. The mechanism of action of peptides was verified by examining the permeabilization of fungal cell membranes, pore formation, and occurrence of oxidative stress and apoptosis. Finally, a potential synergism of the tested peptides with conventional antifungal drugs was analyzed.

## 2. Materials and methods

### 2.1. Peptides

Peptides ΔM3 (NFFKRIRRAGKRIRKAIISA) and ΔM4 (NFFKRIRRAWKRIWKWIYSA), both synthesized by the solid-phase method, were purchased from GenScript (Piscataway, NJ, United States). Purity of the peptides was verified by analytical high performance liquid chromatography (HPLC) and MALDI-TOF mass spectrometry and was determined as >95% for both peptides.

### 2.2. Circular dichroism

Peptides at concentration of 1 mg/ml, diluted in 10 mM HEPES pH 7.0 or PBS, without or with 2,2,2-trifluoroethanol (TFE, Sigma-Aldrich, St Louis, MO, United States) at concentrations in the range of 5–25% (commonly used to stabilize secondary structure of polypeptides), and diluted in 10 mM HEPES pH 7.0 with 30 mM SDS (BioShop Canada Inc., Burlington, Canada) (to mimic anionic environment) were prepared. Peptides diluted in 10 mM HEPES pH 7.0 with NaCl at concentrations in the range of 50–200 mM were used to analyze the effect of salt ions on the peptide secondary structure. Circular dichroism (CD) measurements were conducted using a J-715 spectropolarimeter (Jasco, Tokyo, Japan) equipped with an F25 temperature control unit (Julabo, Seelbach, Germany) in quartz cuvettes of 0.01 cm path-length (Hellma, Jena, Germany) at 30°C. The spectra were recorded in the range of 195–250 nm with 1 nm data pitch, 20 nm/min scanning speed, 4 s integrating time, and 2 nm bandwidth and averaged over five acquisitions. All spectra were corrected for the effect from the buffer and all measurements were converted to molar residual ellipticity units. Secondary structure content was determined using JSSE program (Jasco) using CD spectra of α-chymotrypsin A, hemoglobin, lysozyme, myoglobin and ribonuclease A as protein references.

### 2.3. Yeast strains and culture conditions

**Figure 1:**
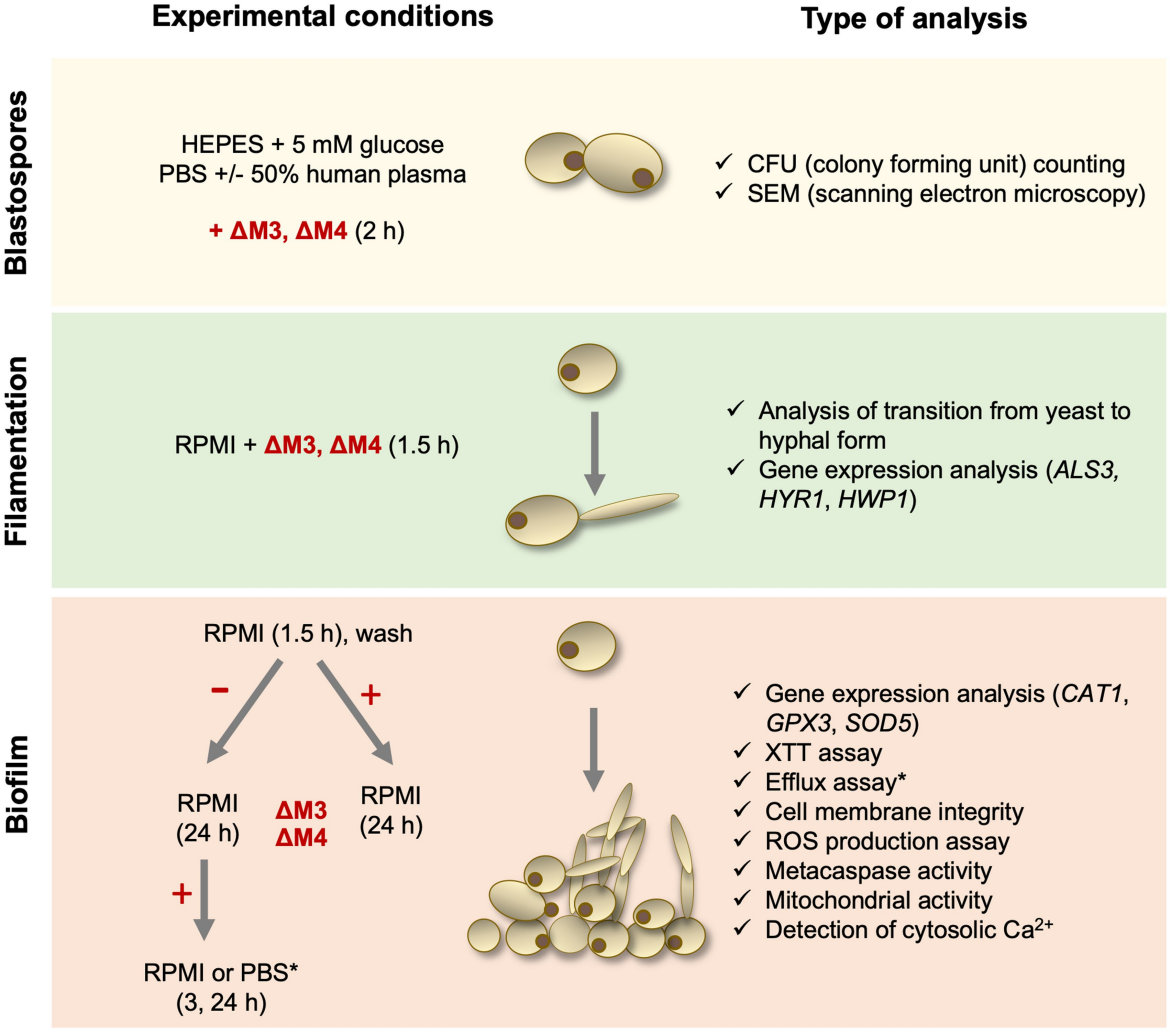
Diagram presenting experimental steps performed in this study.

*Candida albicans* strain 3147 (ATCC^®^ 10231™), *Candida tropicalis* strain T1 (ATCC^®^ MYA-3404™), *Candida parapsilosis* strain CDC 317 (ATCC^®^ MYA-4646™) and *Candida glabrata* strain CBS138 (ATCC^®^ 2001™) were purchased from the American Type Culture Collection (ATCC) (Manassas, VA, United States). Yeast cells (5 × 10^5^/ml at the start) were routinely cultured in YPD medium (1% yeast extract, 2% soybean peptone and 2% glucose) (Sigma-Aldrich) for 18 h at 30°C with shaking (170 rpm) using an orbital rotary shaker MaxQ 6000 (ThermoFisher Scientific, Waltham, MA, United States). Before each experiment, fungal cells were washed three times with the appropriate buffer – phosphate buffered saline pH 7.4 (PBS; Biowest, Nuaillé, France) or 10 mM HEPES pH 7.0 with 5 mM glucose (HEPES/glucose) – and optical density at 600 nm (OD) was measured to estimate the number of cells. To determine the number of colony forming units (CFUs) per ml after treatment with peptide, fungal cells were cultured on solid YPD medium containing 1.5% agar for 24 h at 30°C. Due to the complex experimental setup of this study, a diagram showing the consecutive steps of the experiments is presented (Figure 1).

### 2.4. Fungicidal activity against *Candida* yeast cells

The analysis of fungicidal activity of ΔM3 and ΔM4 peptides was performed as previously described (Bochenska et al., 2016; van Dijck et al., 2018) with minor modifications. Briefly, 1 × 10^5^ yeast cells of *C. albicans* were incubated in 100 μl of ΔM3 or ΔM4 solution at concentrations in the range of 0.4–100 μM in 10 mM HEPES pH 7.0, 10 mM HEPES pH 7.0 with 5 mM glucose, PBS or PBS with 50% of human plasma for 2 h at 30°C with shaking (110 rpm) in Eppendorf tubes. Additionally, cells from each strain were incubated in the buffer without peptides under the same conditions. After incubation, 10 μl of the cell suspensions undiluted and diluted (10 and 100-fold) with PBS or sterile water were plated to the YPD agar dishes for determination of CFUs. Similar experiments with yeast cells of *C. parapsilosis*, *C. tropicalis* or *C. glabrata* were performed in 10 mM HEPES pH 7.0 with 5 mM glucose with peptides in the same range of concentrations. The experiments were performed independently at least three times in triplicates. The minimal fungicidal concentrations – MFC_90_ and MFC_99.9_ that cause mortality of 90 or 99.9% of fungi, respectively, were determined.

### 2.5. Analysis of cell surface morphology of *Candida albicans* yeast cells by scanning electron microscopy (SEM)

*Candida albicans* yeast cells (1 × 10^6^/ml) obtained after 16 h of growth in YPD medium were incubated for 2 h at 30°C in a solution of peptides ΔM3 (1.6 μM) and ΔM4 (3.1 μM) in HEPES/glucose. Cells incubated under the same conditions, but without peptides, served as an untreated control. The preparation of the cells for observation with scanning electron microscopy (SEM) was performed similarly to that described by Staniszewska et al. (2013) with minor modifications. Briefly, *C. albicans* cells were fixed overnight at 4°C in 2.5% glutaraldehyde (Sigma-Aldrich) on round coverslips (⌀13 mm, Bionovo, Legnica, Poland) precoated with 0.001% poly-L-lysine (Sigma-Aldrich), and then washed five times with 0.1 M phosphate buffer, pH 7.2. The further steps included incubation of specimens in 2% osmium tetroxide (Sigma-Aldrich) for 2 h at room temperature and dehydration in a series of ethanol solutions (Linegal Chemicals, Poland), starting from 10 min twice in 50 and 70%, followed by 5 min twice in 95% and 1 min twice in 100% ethanol solution. The samples were then dehydrated with acetone (Merck, Darmstadt, Germany) twice for 30 s, dried by the critical point method in liquid CO_2_ using an E300 critical point dryer (Quorum Technologies, East Sussex, United Kingdom) and coated with gold in a vacuum evaporator JEOL JFC-1100E (JEOL Ltd., Tokyo, Japan). Visualization was performed at an accelerating voltage of 20 kV with the Hitachi S-4700 scanning electron microscope (Hitachi, Tokyo, Japan) in the Laboratory of Scanning Electron Microscopy and Microanalysis, Institute of Geological Sciences, Jagiellonian University, Krakow, Poland.

### 2.6. Microscopic analysis of *Candida albicans* transition from yeast to hyphal form

*Candida albicans* yeast cells (1 × 10^5^ per well) were suspended in 100 μl of RPMI 1640 medium (Biowest) containing peptides at concentrations in the range of 3.1–50 μM and 0.8–6.2 μM for ΔM3 and ΔM4, respectively, and placed in triplicates into the wells of 96-well black/clear flat bottom polystyrene high binding microplate (Corning Inc., Corning, NY, United States). Fungal cells were also suspended in medium without peptide as a control of the correct morphological transition. The incubation was carried out at 37°C in an atmosphere of 5% CO_2_ and 95% humidity for 90 min without shaking. Subsequently, fungal cells were stained with Calcofluor White (Sigma-Aldrich) at a final concentration of 1 μg/ml and Sytox Orange (ThermoFisher Scientific, Waltham, MA, United States) at a final concentration of 1 μM for 10 min in the dark. *C. albicans* cells were visualized using an Olympus IX73 microscope (Olympus, Tokyo, Japan) equipped with a Hamamatsu Orca Spark camera (Hamamatsu, Hamamatsu City, Japan) and a UPLXAPO60XO lens (Olympus), and the images were analyzed using Olympus CellSens Dimension 3.1 software. To determine the length of the hyphae, three images were taken in each well (40 × magnification) and for each image the length of the hyphae was measured for 20 cells.

### 2.7. Gene expression analysis

The expression of genes characteristic for the filamentous form of *C. albicans* – encoding agglutinin-like protein 3 (*ALS3*), hyphally regulated cell wall protein 1 (*HYR1*) and hyphal wall protein 1 (*HWP1*) was analyzed after the isolation of total RNA from cells prepared as follows. Yeast cells (2 × 10^7^) were seeded in plates (⌀10 cm) with 10 ml of RPMI medium in the presence of ΔM3 (at concentrations of 12.5 μM and 25 μM), ΔM4 (at concentrations of 1.2 μM and 2.1 μM) and without peptide. Two plates were prepared for each sample. After 90 min of incubation at 37°C in an atmosphere of 5% CO_2_ and 95% humidity, cells were harvested, washed with PBS and frozen in liquid nitrogen. The expression of genes associated with the generation of oxidative stress (encoding catalase – *CAT1*, glutathione peroxidase – *GPX3*, superoxide dismutase 5 – *SOD5*) was analyzed in mature biofilms. Briefly, yeast cells (5 × 10^7^) were seeded in plates (⌀10 cm) with 10 ml of RPMI medium and incubated for 24 h at 37°C in an atmosphere of 5% CO_2_ and 95% humidity. Subsequently, the medium was changed to a fresh medium supplemented with 50 μM ΔM3, 6.2 μM ΔM4 or without peptide and the biofilms were incubated for 3 h at 37°C. Two plates were prepared for each sample. The concentration chosen for the analysis of gene expression resulted from the need to perform experiments on living cells in order to obtain undegraded mRNA. Cells were prepared for RNA isolation as described below.

Total RNA from cells was isolated with Tri Reagent (Merck Millipore, Burlington, MA, United States) according to a standard procedure preceded by cell homogenization in the Precellys^®^ Evolution tissue homogenizer (Bertin Technologies SAS, Montigny-le-Bretonneux, France). cDNA was synthesized with the M-MLV Reverse Transcriptase kit (Promega, Madison, WI, United States) according to the manufacturer’s instructions. The expression of the selected genes was quantified with the QuantStudio™ 3 Real-Time PCR System (ThermoFisher Scientific). cDNA amplification was performed at an annealing temperature of 58°C using Kappa SYBR Green Master Mix (Merck Millipore) with specific primers (Table 1). Some primers were projected and verified with Primer3, OligoAnalyzer, and BLAST software. Relative gene expression was determined using the 2^-ΔΔCt^ method normalized to the expression of the *ACT1* housekeeping gene.

**Table 1:** The list of primers used for quantification of genes for: *ACT1* – actin; *ALS3* – agglutinin-like protein 3; *CAT1* – catalase; *GPX3* – glutathione peroxidase; *HYR1* – hyphally regulated cell wall protein 1; *HWP1* – hyphal wall protein 1; *SOD5* – superoxide dismutase 5.

Gene	Primer forward 5′ → 3′	Primer reverse 5′ → 3′	Reference
*ACT1*	GATTTTGTCTGAACGTGGTTAACAG	GGAGTTGAAAGTGGTTTGGTCAATAC	Wolak et al. (2015)
*ALS3*	TGCTGGTGGTTATTGGCAAC	GTCGCGGTTAGGATCGAATG	Bartnicka et al. (2019)
*CAT1*	TGGTGGTGAATTAGGTTCTGC	GTGAGTTTCTGGGTTTCTCTT	Wolak et al. (2015)
*GPX3*	TTCACTCCACAATACAAAGGT	TTCATTAGTTCCAGGTTCTTG	This work
*HYR1*	TTGTTTGCGTCATCAAGACTTTG	GTCTTCATCAGCAGTAACACAACCA	Tsang et al. (2012)
*HWP1*	TCAACTGCTCAACTTATTGCT	GCTTCCTCTGTTTCACCTTG	Bartnicka et al. (2019)
*SOD5*	ATCTTACATTGGCGGTTTAT	GACCATTTACTACTGCTCTCTCA	This work

### 2.8. Formation of biofilms

*Candida albicans* biofilms were formed generally in 96-well black/clear flat bottom polystyrene high binding microplate (Corning Inc.) after seeding the cells (1 × 10^5^) in 100 μl of RPMI 1640, followed by incubation for 90 min at 37°C in an atmosphere of 5% CO_2_ and 95% humidity, without shaking to allow cell adhesion. Then non-adherent cells were removed by gentle washing with 200 μl of PBS and 100 μl of fresh RPMI 1640 were added to sessile *C. albicans* cells for further incubation under the same conditions for 24 h to allow formation of the biofilm.

### 2.9. Antibiofilm assay

To determine the impact of peptides on the formation of biofilm, adherent cells, gently washed as described above, were incubated with peptides at a concentration range of 0.4–100 μM in 100 μl of RPMI medium for 24 h at 37°C in an atmosphere of 5% CO_2_ and 95% humidity. After this time biofilms were washed, and the biofilm metabolic activity was assessed. To examine the effect of peptides on the eradication of the 24-h developed biofilm, a method previously described (Roscetto et al., 2018) was used. The 24-h biofilms (prepared as described above) were washed twice with 200 μl of PBS and then further incubated for 24 h at 37°C in an atmosphere of 5% CO_2_ and 95% humidity, with 100 μl of RPMI 1640 medium (control) or with ΔM3 or ΔM4 at a concentration ranging from 1.6 μM to 100 μM in the same medium. After incubation, the biofilms were washed twice with 200 μl of PBS and the metabolic activity of *C. albicans* was measured using the XTT reduction test. All experiments were carried out in triplicate.

### 2.10. Analysis the metabolic activity of *Candida albicans* cells

To determine the viability of *C. albicans,* the 2,3-bis(2-methoxy-4-nitro-5-sulfo-phenyl)-2H-tetrazolium-5-carboxanilide (XTT) (ThermoFisher Scientific) reduction assay is used as a routine tool for quantitative measurement of fungal metabolic activity, growth and response to antifungal treatment (van Dijck et al., 2018). The tests were performed as described (Kulig et al., 2022) with some modifications. Briefly, 100 μl of RPMI 1640 medium without phenol red (Biowest) and 50 μl of XTT solution (1 mg/ml; Invitrogen, Waltham, MA, United States), freshly activated with phenazine methosulfate (5 μg/ml; Sigma-Aldrich) in the same medium, were added to *C. albicans* cells. After 40 min of incubation at 37°C in the dark, 100 μl of supernatants were transferred to the wells of a new 96-well microplate (Sarstedt, Nümbrecht, Germany). The absorbance of the formazan product was measured spectrophotometrically at 450 nm using a Synergy H1 microplate reader (BioTek Instruments, Winooski, VT, United States).

### 2.11. Analysis of the cell membrane integrity in the mature biofilm

To determine the impact of peptides on the integrity of *C. albicans* membranes in the mature biofilm, two types of permeabilization assays were performed. In one of them, 24-h biofilms were washed with 200 μl of PBS and then incubated with peptides diluted in RPMI at concentrations in the range of 12.5 μM to 100 μM (ΔM3) and 3.1 μM to 12.5 μM (ΔM4) for 24 h at 37°C in an atmosphere of 5% CO_2_ and 95% humidity. After incubation, the biofilms were stained with Sytox™ Orange Nucleic Acid Stain (Invitrogen) at a final concentration of 1 μM in the dark for 5 min and visualized using an Olympus IX73 microscope. All images were taken and presented using the same parameters (exposure time, spectrum range), which enabled comparison of the fluorescence of the dye between different preparations. The photographs were taken in the form of the Z-stacs and subjected to the process of 3D deconvolution.

The second type of experiment was performed as described previously by Do Nascimento Dias et al. (2020). The 24-h biofilms were washed three times with 200 μl of PBS and then incubated in 100 μl of PBS for 2 h at 37°C. After this time, 100 μl solution of Rhodamine 6G (Sigma-Aldrich) in PBS (final dye concentration – 10 μM) was added to the wells and the biofilms were incubated for additional 30 min under the same conditions. To remove non-internalized dye, the wells were washed four times with PBS. Then 100 μl of peptide solutions were added to biofilms at concentrations in the range of 1.6 μM to 100 μM in PBS and incubated for 24 h at 37°C in an atmosphere of 5% CO_2_ and 95% humidity. The untreated biofilms served as controls. After incubation, the supernatants were transferred to the new black microplate and the fluorescence intensity was measured using a Synergy H1 microplate reader with excitation at 550 nm and emission at 580 nm.

### 2.12. Metacaspase activity

Metacaspase activity was determined using the fluorescent method described previously (Shirazi and Kontoyiannis, 2015) with minor modifications. Briefly, the 24-h biofilm of *C. albicans* was formed in 96 well glass-like microplate (Cellvis, Sunnyvale, CA, United States) and incubated for 24 h at 37°C with ΔM3 or ΔM4 at concentrations in the range of 3.1–12.5 μM or 12.5–100 μM, respectively, diluted in 100 μl of RPMI 1640. After incubation, the biofilms were washed three times with 200 μl of PBS and stained with CaspACE™ FITC-VAD-FMK *In Situ* Marker (Promega, Madison, WI, United States) at a final concentration of 10 μM for 1 h at room temperature in the dark. The biofilms were washed four times with PBS and the activity of metacaspase was analyzed using the Olympus IX73 microscope.

### 2.13. ROS production assay

The generation of intracellular reactive oxygen species (ROS) by *C. albicans* was determined using a method based on the measurement of the fluorescence of oxidized dihydrorhodamine 123 (DHR 123). The 24-h biofilms of *C. albicans*, formed as described above, were washed with PBS and then incubated with 100 μl of RPMI 1640 medium (untreated control), or solutions of ΔM3 (50 μM), ΔM4 (6.2 μM), or amphotericin B (2.7 μM; positive control) (Biowest) prepared in RPMI 1640 medium. For the analysis of ROS production, the concentration of peptides corresponding to the apoptotic effect observed with the activity of metacaspases was selected. The incubation was carried out for 3 h at 37°C. Biofilms were then washed with PBS and incubated with non-fluorescent DHR 123 (Invitrogen) at a final concentration of 10 μM for 30 min at 37°C. The fluorescence of oxidized DHR 123 (rhodamine 123) was measured with excitation at 488 nm and emission at 525 nm using a Synergy H1 microplate reader.

### 2.14. Determination of mitochondrial activity

The 24-h biofilms of *C. albicans* were incubated with 100 μl of ΔM3 (50 μM) or ΔM4 (6.2 μM) and amphotericin B (2.7 μM; positive control) solutions in RPMI 1640 medium for 3 h at 37°C. After that time, biofilms were washed with 200 μl of PBS and incubated for 30 min at 37°C with MitoTracker Green TM dye (Invitrogen) at final concentration of 500 nM, used to detect the change in total mitochondrial mass, and MitoTracker Orange (500 nM), used to detect the change in mitochondrial activity. Mitochondria were imaged in the FITC and TRIC channel, using the Olympus IX73 microscope.

### 2.15. Detection of cytosolic Ca^2+^

Accumulation of cytosolic Ca^2+^ was measured with the Fura-2 Calcium Flux Assay Kit (Abcam, Cambridge, Great Britain). The 24-h biofilms of *C. albicans* were incubated for 3 h with 100 μl of ΔM3 (25 μM and 50 μM) or ΔM4 (6.2 μM and 12.5 μM) and H_2_O_2_ (10 mM; positive control) solutions prepared in RPMI 1640. The concentration of peptides was chosen close to those for which the effect of apoptosis was obtained in experiments analyzing the activity of metacaspases. After incubation, biofilms were washed with Hanks Balanced Salt Solution (Biowest) with Pluronic^®^ F127 Plus (Abcam). Then 100 μl of Fura-2 AM solution was added to the biofilms and incubated for 1 h at room temperature. The fluorescence was then measured using a Synergy H1 microplate reader with excitation at 340 nm and emission at 510 nm.

### 2.16. Actions of ΔM3 and ΔM4 peptides mixed with conventional antifungal drugs

The following concentrations of peptides and antifungal drugs were used for the assay: ΔM3 0.4–50 μM, ΔM4 0.4–3.1 μM, amphotericin B 0.2–1.6 μM, fluconazole 0.2–1.6 μM and caspofungin 0.003–0.2 μM (the latter two from Sigma-Aldrich). The combinatory effects of each peptide and each conventional antimycotic drug were tested by the previously described checkerboard method (Guo et al., 2008; Do Nascimento Dias et al., 2020) with minor modification. *C. albicans* cells (3 × 10^2^) were suspended in 100 μl of RPMI 1640 medium, containing peptide, drug, or their combination, in wells of Falcon^®^ 96-well clear flat bottom TC-treated culture microplate (Corning). Fungal cells only in RPMI 1640 medium without peptides and drugs served as a control. Incubation was carried out for 24 h at 37°C in an atmosphere of 5% CO_2_ and 95% humidity without shaking. After this time, cell viability was measured using an XTT reduction assay. The analysis was performed in biological triplicate. For each combination, Fractional Inhibitory Concentration Index (FICI) was determined using the following formula:

FICI = FICa + FICb

Where FICa is the ratio of IC_90_ value for the compound a in combination to the IC_90_ value of the agent a alone; FICb is the ratio of IC_90_ value for compound b in combination to the IC_90_ value of the agent b alone.

The FICI ≤0.5 was interpreted as a synergistic effect, 0.5< FICI ≤1.0 as additive, 1.0< FICI ≤2.0 as indifferent and FICI >2.0 as antagonism.

### 2.17. The effect of ΔM3 and ΔM4 on human dermal fibroblasts

The toxicity of the peptides was analyzed on primary human dermal fibroblasts (HDF) obtained from Cell Applications Inc. (San Diego, CA, United States). Cells (1 × 10^5^), seeded in a microplate, were treated with peptides with a concentration in the range of 1.6–100 μM for 24 h in culture HDF basal medium (Cell Applications Inc.). After this time, cell viability was assessed with the Alamar blue test (ThermoFisher Scientific). Briefly, after medium removal, 100 μl of Alamar blue reagent were added and after 1 h of incubation the fluorescence was measured with excitation at 560 nm and emission at 590 nm with Synergy H1 microplate reader.

### 2.18. Statistical analysis

Statistical analysis was performed with the GraphPad Prism 8 software (GraphPad Software, San Diego, CA, United States). To evaluate the significance between groups, the t-test or one-way ANOVA with Dunnett’s multiple comparison *post hoc* test was performed. The results were considered statistically significant at value of *p* < 0.05: *p ≤ 0.05, **p < 0.01, ***p < 0.005, ****p < 0.0001 or not statistically significant (ns) for *p* > 0.05.

## 3. Results

### 3.1. Synthetic peptides ΔM3 and ΔM4 possess fungicidal activity

**Figure 2:**
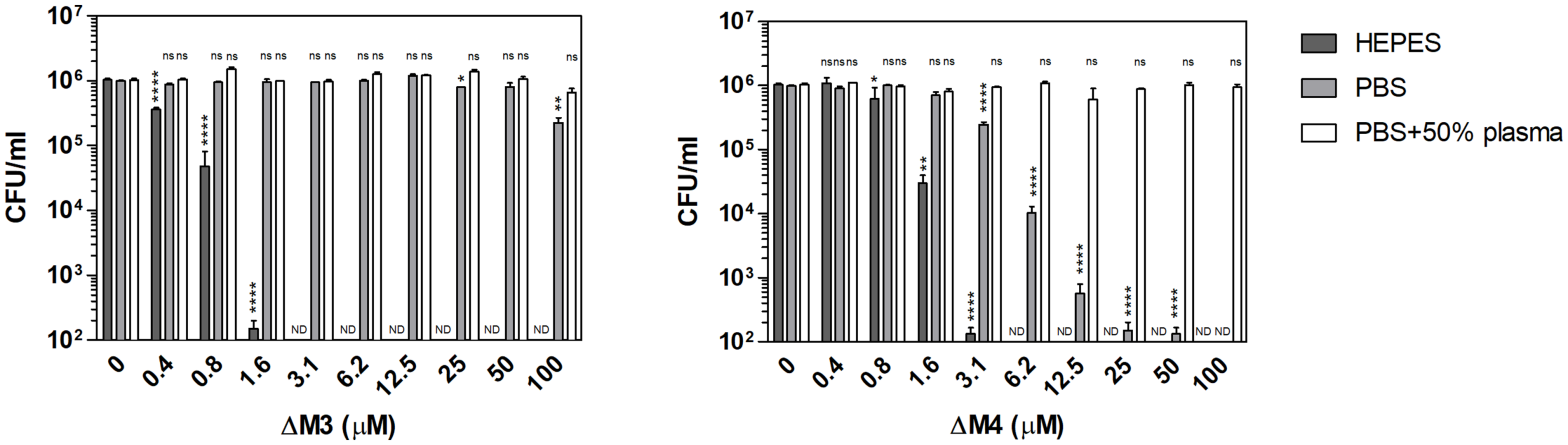
Fungicidal activity of ΔM3 and ΔM4 against *C. albicans* under various conditions. *C. albicans* yeast cells (10^5^/100 μl) were incubated with ΔM3 and ΔM4 at concentrations in a range of 0.4–100 μM for 2 h in 10 mM HEPES, PBS, or PBS with 50% human plasma. The cell suspensions were plated on solid YPD medium. After 24 h of cultivation at 30°C, the number of CFUs was determined. The experiment was performed in triplicate. Graphs present mean values ± SD from representative results (*n* = 4). For statistical analysis one-way ANOVA with Dunnett’s multiple comparisons *post hoc* test was used (*p < 0.05, **p < 0.005, **** *p* < 0.0001, ns > 0.05 vs. untreated cells). A value of 10^2^ CFU/ml on the y-axis was the limit of quantification. Values that were below this limit were marked ND.

The interaction between antimicrobial peptides and the pathogen cell wall plays an important role in their fungicidal activity. The achieved effect depends, among others, on environmental factors such as pH, the presence of salt or the presence of substances that can change the hydrophobicity of the peptide (Kandasamy and Larson, 2006). To test the influence of the environment on the fungicidal activity of two cationic peptides, ΔM3 and ΔM4, the viability of *C. albicans* cells was analyzed in the presence of some commonly used media: HEPES buffer supplemented with 5 mM glucose (HEPES/glucose) and PBS without or with 50% human plasma. The peptides showed different activity against *Candida* cells depending on the conditions used (Figure 2). The ΔM4 peptide, which is more hydrophobic, was active in the presence of PBS or HEPES/glucose, while the ΔM3 peptide was active only in the HEPES/glucose. Both peptides, ΔM3 and ΔM4 incubated in HEPES/glucose had a strong fungicidal effect against *C. albicans* after 2 h of incubation at concentrations of 1.6 μM and 3.1 μM, respectively. Peptide ΔM4 in PBS caused a weaker effect compared to that in HEPES/glucose.

**Figure 3:**
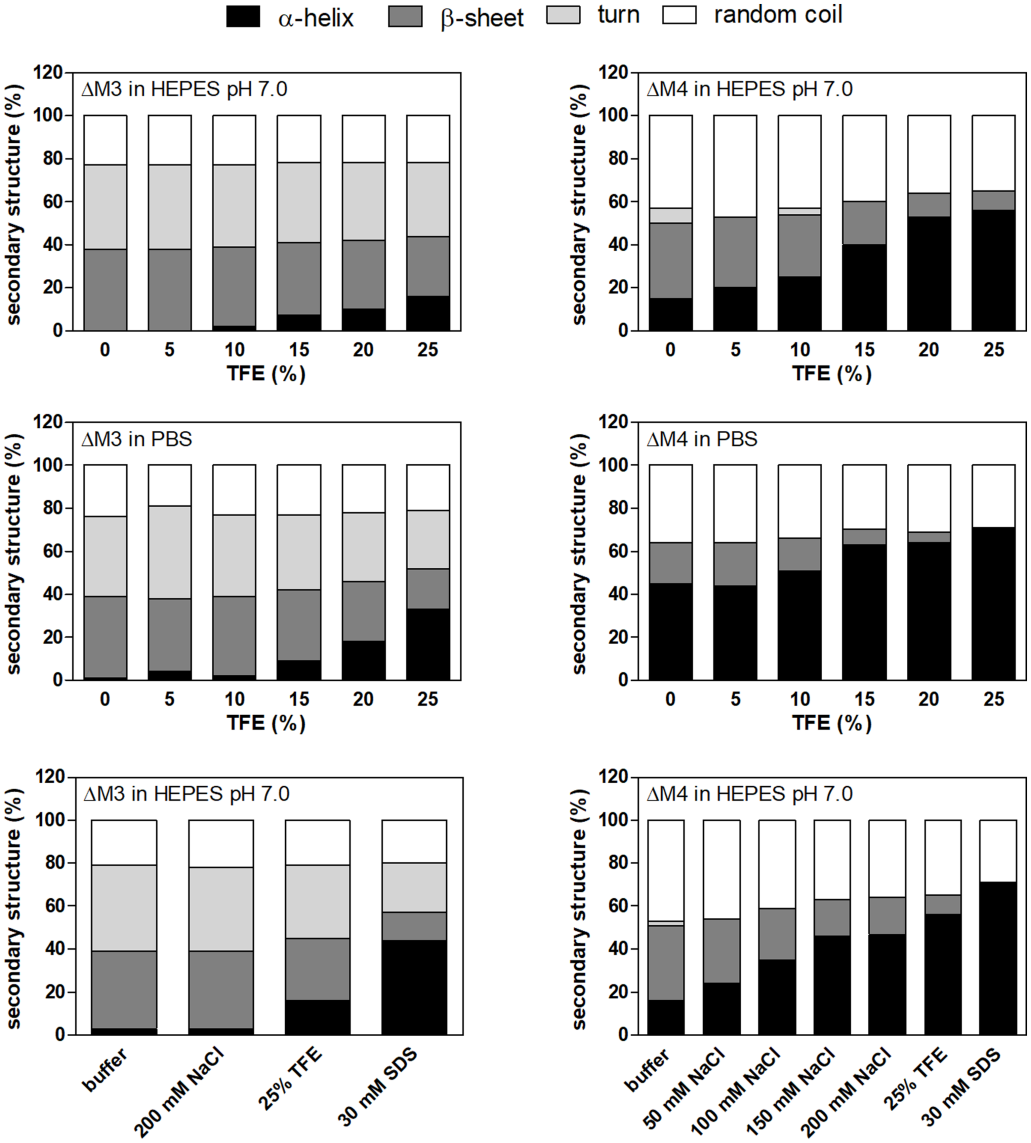
The secondary structure of peptides in different solutions analyzed by circular dichroism (CD) measurements. ΔM3 and ΔM4 peptides (both at a concentration of 1 mg/ml) were prepared in 10 mM HEPES pH 7.0 or PBS. Trifluoroethanol (TFE) at a final concentration in a range of 5–25% was used to enhance secondary structure stability. To check the effect of NaCl on the secondary structure of the peptides, samples were prepared in 10 mM HEPES pH 7.0 with NaCl (50–200 mM). Buffer supplemented with 30 mM SDS was used to mimic microbial membrane. CD spectra of peptides were measured in the range of 195–250 nm and averaged over five acquisitions. Secondary structure content was determined using JSSE program (Jasco) using CD spectra of α-chymotrypsin A, hemoglobin, lysozyme, myoglobin and ribonuclease A proteins as a reference.

To clarify the observed effect of both peptides on fungal cell viability, peptide structural studies were performed using circular dichroism measurements (Figure 3; Supplementary Figure S1). In this analysis, we used TFE (2,2,2-trifluoroethanol) as a cosolvent that promotes the stability of the peptide helical structure (Maisetta et al., 2008; Górecki et al., 2015). Nonetheless, upon contact with the fungal cell surface, hydrophobic interactions may also be primary forces driving the formation of the secondary peptide structure. As demonstrated in Figure 3, ΔM3 peptide in buffer solution, containing TFA at increasing concentrations, was less susceptible to adopt a helical structure compared to ΔM4, even in the presence of SDS, popularly used in such tests. The pH changes of the buffers used (pH 7.0 versus pH 7.4) did not exert a significant effect on the peptide helical structure. However, the structural preferences of both peptides differ significantly in the presence of salt, which is particularly relevant for experiments conducted in PBS and in RPMI 1640 medium. The presence of salt in a concentration-dependent manner enhanced the helicity of ΔM4 but had no effect on structural properties of ΔM3. These observations may explain the results obtained for the peptide treatment of fungal cells and their killing effects. Furthermore, supplementation with human plasma resulted in a loss of activity of both peptides, as no significant changes in fungal viability were observed within the range of peptide concentrations tested. Such reduction in the antibacterial activity of the peptides was often observed due to their nonspecific binding to the plasma proteinous components (Sovadinová et al., 2021).

Since the investigated peptides showed the best fungicidal properties against *C. albicans* in HEPES/glucose, the antifungal activity of these compounds against different *Candida* species was tested under the same conditions. Both peptides showed high efficiency against *C. tropicalis* and *C. parapsilosis,* reaching values of minimal fungicidal concentrations (MFCs) in the same range as those determined for *C. albicans* (Table 2). The values of MFC_90_ and MFC_99.9_ were of 0.8 μM and 1.6 μM, respectively, for ΔM3, and of 1.6 μM and 3.1 μM, respectively, for ΔM4. *C. glabrata* showed higher resistance to these peptides. For the ΔM3 peptide, the value of MFC_99.9_ was not determined within a range of peptide concentration used, while for ΔM4, which presented greater antifungal activity, MFC_99.9_ was estimated to be 50 μM. Nevertheless, these observations indicate that both peptides under these conditions may be potential antifungal agents against yeast-like forms of *Candida* species, especially *C. albicans*, *C. parapsilosi*s and *C. tropicali*s.

**Table 2:** Minimal fungicidal concentrations (MFC_90_ and MFC_99.9_) of ΔM3 and ΔM4 against yeast cells of *Candida* species.

Yeast	ΔM3		ΔM4	
	MFC_90_ (μM)	MFC_99.9_ (μM)	MFC_90_ (μM)	MFC_99.9_ (μM)
*C. albicans* strain 3147	0.8	1.6	1.6	3.1
*C. tropicalis* strain T1	0.8	1.6	1.6	3.1
*C. parapsilosis* strain CDC 317	0.8	1.6	1.6	3.1
*C. glabrata* strain CBS138	1.6	ND	1.6	50

MFC_90_ and MFC_99.9_ were determined against 10^5^ planktonic cells of *C. albicans*, *C. tropicalis*, *C. parapsilosis* and *C. glabrata* incubated for 2 h at 30°C with peptides in the concentration range of 0.4–100 μM, diluted in 10 mM HEPES pH 7.0 with 5 mM glucose, using the CFU assay. The experiment was carried out three times independently. All strains were obtained from ATCC. MFC_90_ and MFC_99.9_ – minimal concentration of peptide that causes mortality of 90 and 99.9% fungal cells, respectively, ND – not detected in the range 0.4–100 μM peptide (over the detection limit).

**Figure 4:**
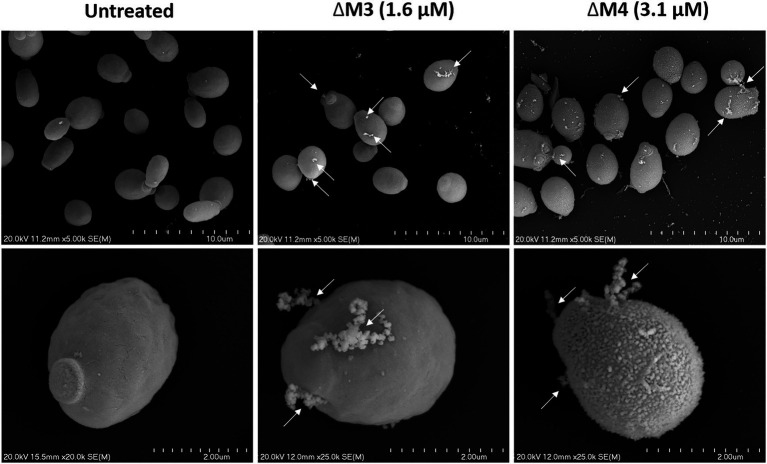
Scanning electron microscopy of the *C. albicans* cell surface after treatment with ΔM3 and ΔM4. *C. albicans* yeast cells (10^5^/100 μl) were incubated with ΔM3 (1.6 μM) or ΔM4 (3.1 μM) in 10 mM HEPES pH 7.0 with 5 mM glucose for 2 h at 30°C, then fixed overnight with glutaraldehyde, dehydrated, coated with gold and examined with a scanning electron microscope HITACHI S-4700. Cells incubated in buffer without peptides served as an untreated control. Arrows indicate surface damage that leads to intracellular material leakage.

The identified effects of ΔM3 and ΔM4 on *C. albicans* cell viability were also confirmed by SEM analysis of their cell surface structure. The yeast cells showed altered cell surface morphology after incubation with peptides (Figure 4). The untreated cells had a smooth surface and normal shape, whereas treatment with peptides at fungicidal concentration in HEPES/glucose buffer resulted in damage of the cell surface with leakage of intracellular content, marked with arrows on the figures. Similar observations were shown in a recent work, in which *C. albicans* cells were treated with the cecropin 4-derived peptide C18 (Sun et al., 2022). The cells treated with ΔM4 showed also a rough surface. These quite distinct changes in the cell surface under the influence of both tested peptides may indicate their differential interaction with the cell wall of *C. albicans.*

### 3.2. The ∆M4 peptide affects the morphological transition of *Candida albicans*

**Figure 5:**
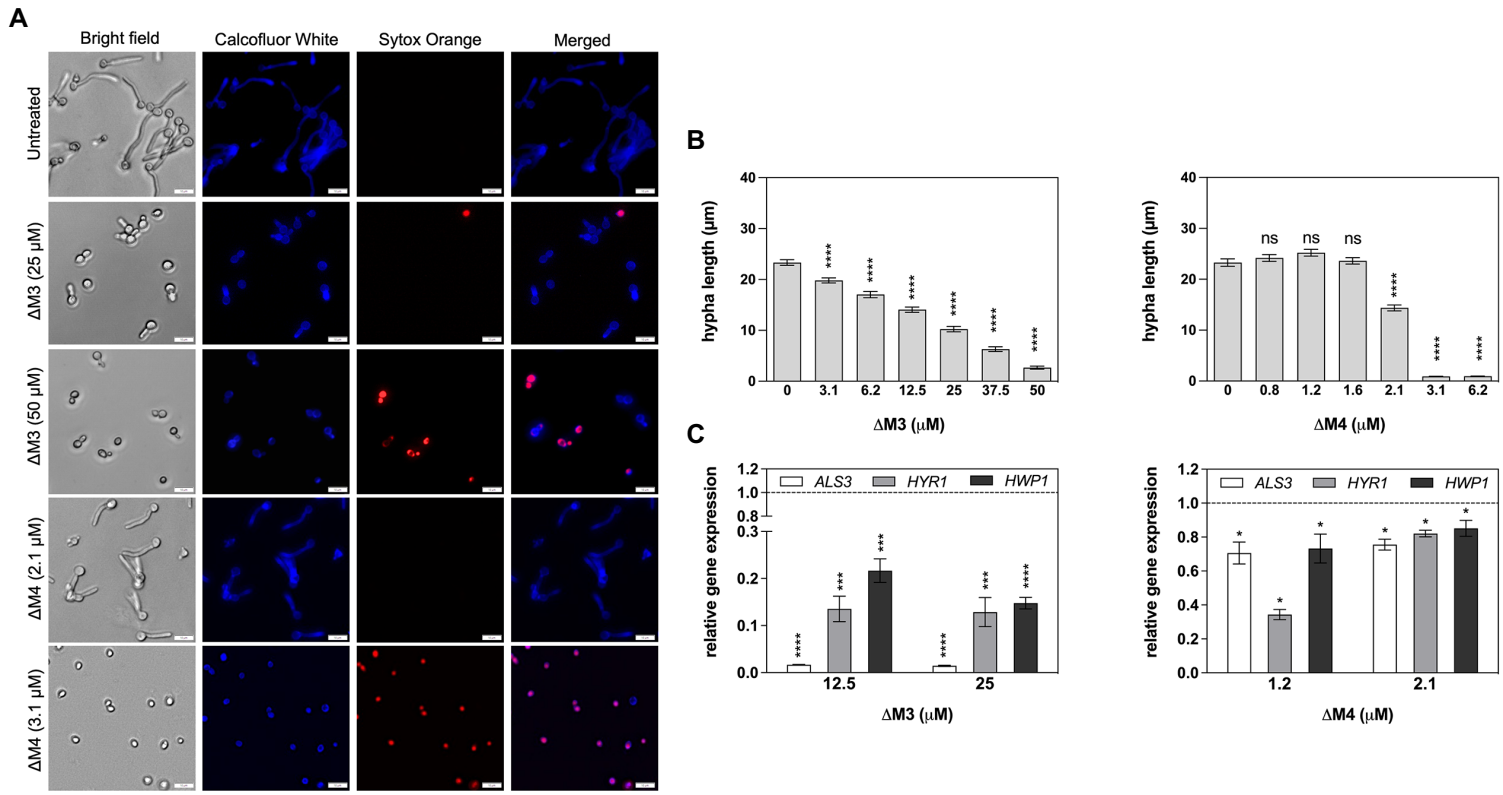
Impact of ΔM3 and ΔM4 on the transition of *C. albicans* from yeast to filamentous form. **(A)** *C. albicans* (10^5^/100 μl) were incubated in RPMI 1640 medium with peptides at concentrations in the range of 3.1–50 μM (ΔM3) or 0.8–6.2 μM (ΔM4) for 90 min at 37°C. The fungi were stained with Calcofluor White (1 μg/ml) or Sytox Orange (1 μM) and visualized using Olympus IX73 microscope. **(B)** The length of the hyphae (*n* = 20) was measured using Olympus CellSens programming. For statistical analysis one-way ANOVA with Dunnett’s multiple comparisons *post hoc* test was used (****p < 0.001). **(C)** The mRNA expression of *ALS3*, *HYR1* and *HWP1* was determined after contact of *C. albicans* cells (2 × 10^6^/ml) with ΔM3 (12.5 μM and 25 μM) and with ΔM4 (1 μM and 2 μM) for 90 min at 37°C. Bars represent the mean value ± SD from at least three independent experiments. A paired Student’s t test was used for statistical analysis. *p < 0.05, ***p < 0.005 and **** *p* < 0.0001 vs. untreated cells (dashed line).

*Candida albicans* is characterized by morphological diversity that allows it to adapt to various environmental conditions to survive, and the transition from yeast to hyphae is essential for host cell colonization by *C. albicans* (Karkowska-Kuleta et al., 2009; Jacobsen et al., 2012). In this study, the effect of both peptides on inhibition of yeast to hyphae transition was investigated using *C. albicans* cells cultured in RPMI 1640 medium that activates filamentation (Figure 5A). The increasing concentration of ΔM3 peptide caused a progressive decrease in cell filamentation. The average length of cell hyphae at peptide concentrations that do not affect cell permeability – 12.5 μM and 25 μM – was approximately 30 and 50% shorter, respectively, compared to that observed in untreated cells (Figure 5B). Incubation of cells with higher concentrations of this peptide (50 μM) led to an increased influx of Sytox Orange dye, indicating permeabilization of the cells. In turn, ΔM4 peptide already at 2.1 μM concentration inhibited the *C. albicans* hypha formation, reducing their length by approximately 30%. However, for concentrations of 3.1 μM and 6.2 μM, ΔM4 completely blocked filamentation, probably killing the cells, as demonstrated by cell permeability test performed with Sytox Orange staining (Figure 5A). The observations of the effect of peptides on fungal morphology were also confirmed by the analysis of the expression of genes associated with hypha formation. Cell treatment with ∆M3 (12.5 μM and 25 μM) significantly decreased the expression of genes encoding the main fungal adhesins: *ALS3*, *HYR1* and *HWP1* compared to untreated cells (Figure 5C), which corroborated the assumption that ΔM3 contributes to the inhibition of hypha formation. However, for fungal cell treatment with ΔM4, only minor changes in expression of these genes were observed, suggesting that ΔM4 is less effective in inhibiting hypha formation by *C. albicans* cells.

### 3.3. Effect of ∆M3 and ∆M4 peptides on *Candida albicans* biofilm

**Figure 6:**
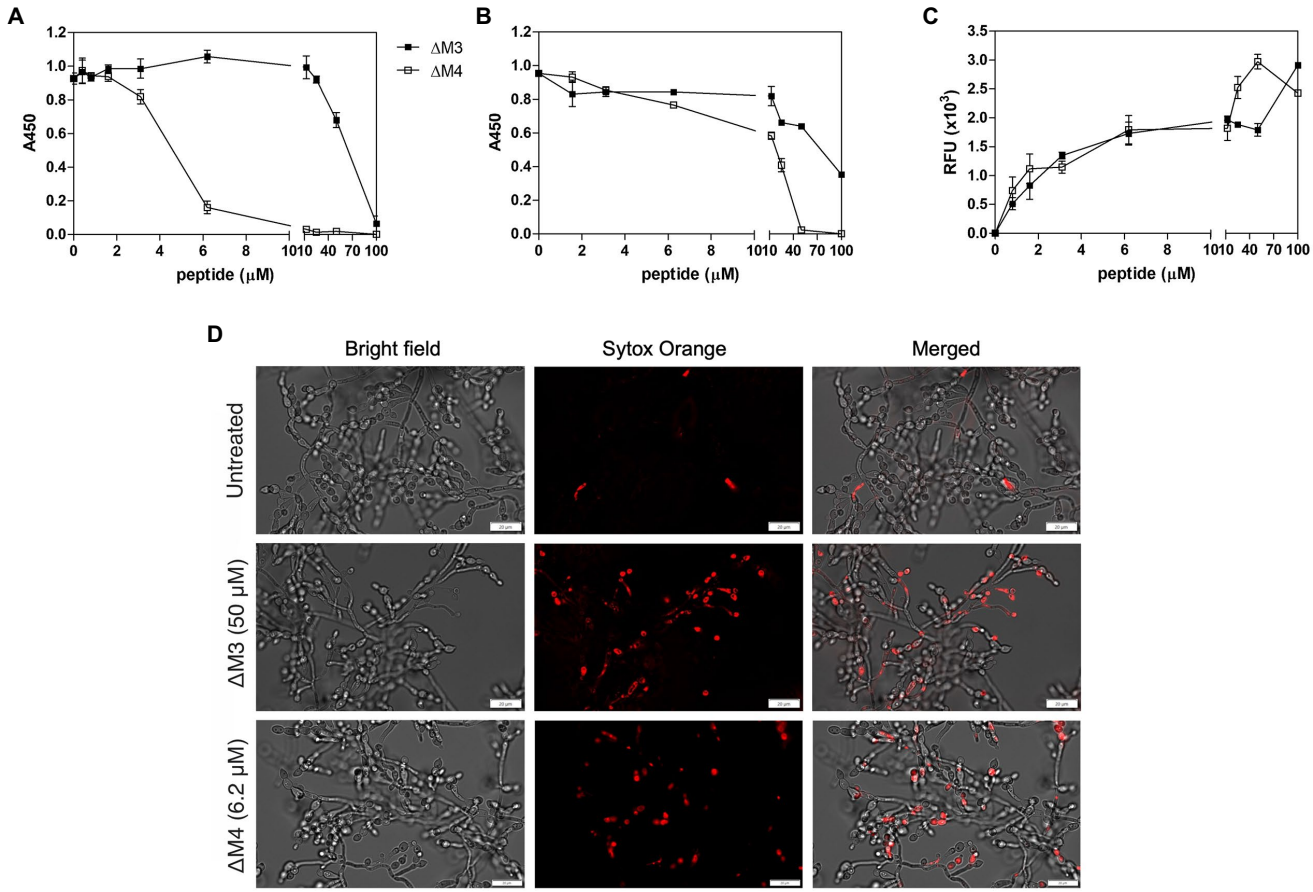
Effect of ΔM3 and ΔM4 on *C. albicans* biofilm. **(A)** The effect of peptides on biofilm formation was performed with *C. albicans* cells (1 × 10^5^/100 μl) incubated in RPMI 1640 medium with peptides at concentrations in a range of 0.4–100 μM for 24 h at 37°C in a microplate wells, after removing unbound cells. The metabolic activity of the biofilm cells was determined using an XTT reduction assay with absorbance measurements at 450 nm (A450). The untreated biofilm served as a control. The mean value ± SD from at least three independent experiments was presented. **(B)** Changes in metabolic activity of *C. albicans* biofilm-forming cells were evaluated using an XTT reduction assay after 24-h biofilm treatment with peptides at concentrations in a range of 0.8–100 μM for 24 h at 37°C. **(C)** Membrane permeability in mature biofilm cells was determined using an adaptation of the Rhodamine G6 efflux assay. The leakage of the fluorescence dye, previously internalized by *C. albicans* cells from mature biofilm, was measured in the supernatants after 24 h of contact of the biofilm with peptides at concentration range of 0.8–100 μM at 37°C. The results of three independent experiments are presented as means ± SD. RFU – relative fluorescence units. **(D)** Representative images of the membrane permeabilization study with Sytox Orange dye. The mature *C. albicans* biofilms, untreated or treated with ΔM3 (50 μM) or ΔM4 (6.2 μM) for 24 h at 37°C, were incubated with 1 μM dye for 5 min in the dark. The biofilms were analyzed using the Olympus IX73 microscope.

The ability to form filaments by *Candida* spp. is not only required for increased virulence but also plays an important role in biofilm formation (Gulati and Nobile, 2016). The potential of the peptides to inhibit biofilm formation was tested after 24 h of incubation at a wide range of peptide concentrations (Figure 6A). The metabolic activity of cells in the biofilms was determined by the XTT reduction assay and compared to biofilm cells not treated with peptides. As expected, the ΔM4 peptide showed a better ability to completely inhibit biofilm formation already at a concentration of 12.5 μM, while ΔM3 was less effective, achieving a similar effect only at a concentration of 100 μM.

Biofilm elimination is an important target in candidiasis, as the formation of this structure is strongly associated with its increased drug resistance (Tsui et al., 2016). The ability of ΔM3 and ΔM4 peptides to eradicate the *C. albicans* biofilm was revealed with the XTT reduction assay. The mature biofilm was more susceptible to ΔM4 than to ΔM3 (Figure 6B). As estimated after 24 h of biofilm incubation in the presence of ΔM4, the metabolism of cells was completely abolished at a concentration higher than 50 μM, while ΔM3 was unable to achieve this effect at this concentration. At a concentration of 100 μM, ΔM3 reduced cell metabolic activity only by 60%.

To confirm the effect of the peptides on *C. albicans* biofilm, cell membrane permeabilization was tested with two different assays. In the Rhodamine G6 efflux assay modified according to Do Nascimento Dias et al. (2020), at the beginning cells were loaded with dye, and after washing and incubation with peptides in glucose-free PBS, the efflux of dye was assessed in the supernatant (Figure 6C). The biofilm was affected by both peptides in a dose-dependent manner, although ΔM4 concentrations above 12.5 μM caused a strongly increased membrane permeabilization, while for ΔM3 it was observed at concentrations above 50 μM. The membrane permeability was also studied using Sytox Orange dye, which stain nucleic acids in cells with altered membranes. The dye load was increased with rising peptide concentration, showing a significant change in cell permeability treated with 6.2 μM ΔM4 and 50 μM ΔM3. Representative images from microscopic analysis showed cell dye load for peptides at these concentrations (Figure 6D). These results correlated with that of the Rhodamine G6 test confirming the influence of peptides on the membrane alteration of *C. albicans* cells forming biofilms; however, ΔM4 was more effective at lower concentrations.

### 3.4. **Δ**M3 and **Δ**M4 peptides induce oxidative stress and pro-apoptotic responses in *Candida albicans* biofilm

**Figure 7:**
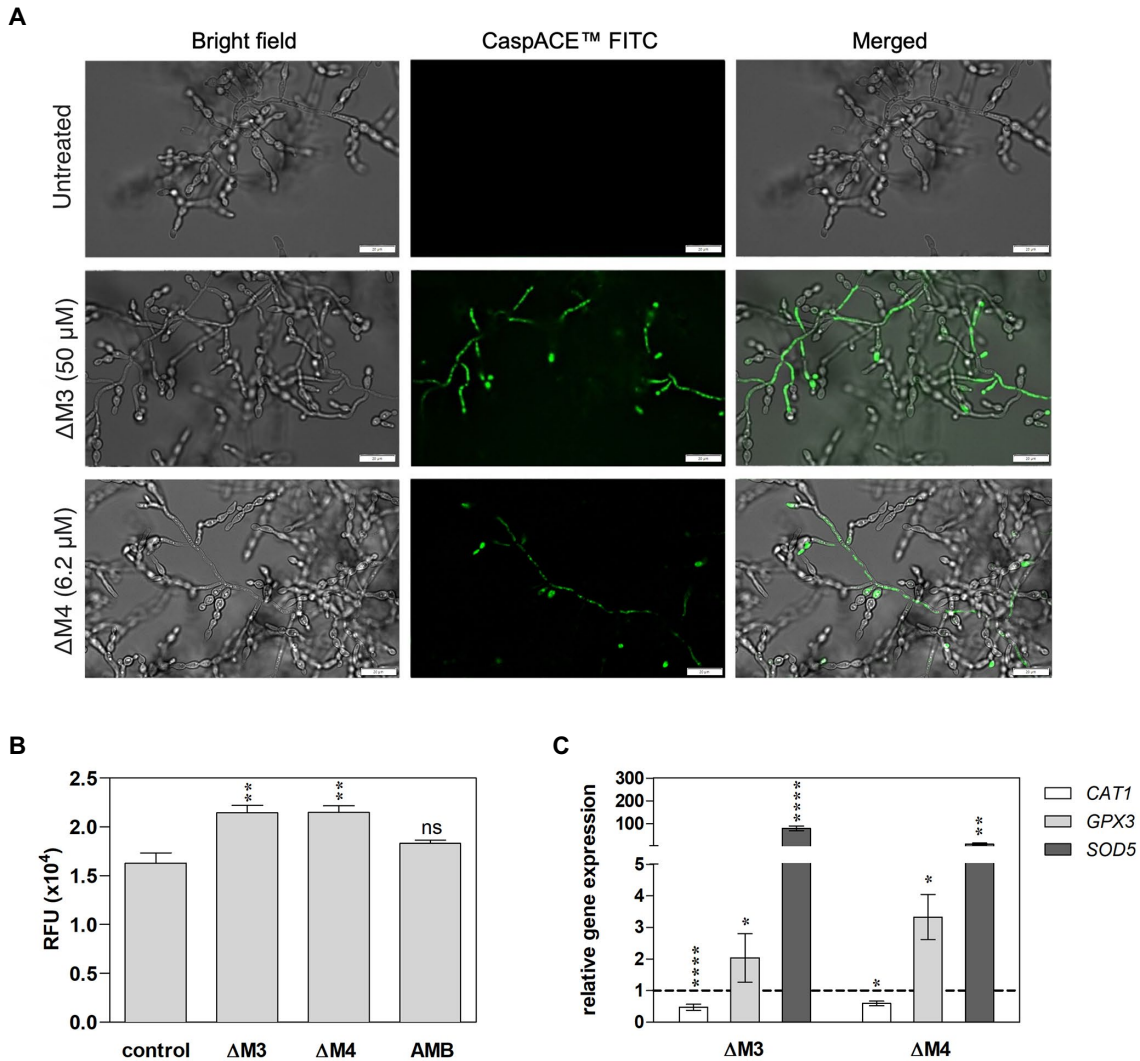
Apoptotic response and oxidative stress induced by ΔM3 and ΔM4 in *C. albicans* mature biofilm. **(A)** *C. albicans* cells (10^5^/ml) were seeded in a 96-well plate and biofilms were grown for 24 h, then peptides were added to the cells in the concentration range of 12.5–100 μM (ΔM3) or 3.1–12.5 μM (ΔM4). The biofilm was grown in the presence of peptides for a further 24 h. The CaspACE ™ FITC-VAD-FMK *In Situ* Marker kit was used to assess metacaspase activation. The marker (10 μM) was added to the biofilms and incubation was carried out for 1 h at 37°C. Biofilms were imaged with an Olympus IX73 microscope and representative images for ΔM3 (50 μM) and ΔM4 (6.2 μM) are presented. **(B)** Intracellular ROS production in mature biofilm by peptides was evaluated with DHR test. The 24-h *C. albicans* biofilms were treated with ΔM3 (50 μM), ΔM4 (6.2 μM) and amphotericin B (2.7 μM) for 3 h at 37°C, washed with PBS and then incubated with DHR 123 (10 μM) for 30 min. The untreated *C. albicans* biofilm served as a control. The fluorescence of oxidized DHR 123 was measured using the BioTek Synergy H1 microplate reader. The graphs present values as mean ± SD (*n* = 3), RFU- relative fluorescence units. Statistical analysis was performed with one-way ANOVA with Dunnett’s multiple comparisons *post-hoc* test. **p < 0.01 **(C)** Analysis of *CAT1*, *GPX1,* and *SOD5* gene expression was performed in mature biofilm cells treated with ΔM3 (50 μM) or ΔM4 (6.2 μM) for 3 h at 37°C. The bars represent the mean values ± SD from at least 3 experiments. A paired Student’s t-test was used for statistical analysis. *p < 0.05, **p < 0.01, ****p < 0.0001 vs. untreated cells (dashed line).

Mechanisms of antifungal activity of peptides are diverse and include not only cell wall disturbances, but also more sophisticated processes associated with the induction of oxidative stress that can lead to cell apoptosis (Vriens et al., 2014; Seyedjavadi et al., 2020). To determine a possible mechanism of *C. albicans* biofilm destruction, the activity of yeast metacaspases was examined in biofilm treated with ΔΜ3 and ΔM4 at different concentrations of peptides. The metascapase activity was detected with CaspACE™ FITC-VAD-FMK *In Situ* Marker, an FITC-marked inhibitor of caspases, which irreversible binds to the enzyme, emitting fluorescence *in situ*. Representative images of microscopic analysis of treated biofilms are shown in Figure 7A. The minimum concentration of peptides, which triggers metacaspase activity in yeast cells and hyphae, was 6.2 μM and 50 μM for ΔM4 and ΔM3, respectively. Pro-apoptotic processes often result in loss of ROS concentration balance in cells and lead to oxidative stress. Here, the effect of ΔΜ3 and ΔM4 peptides on the induction of oxidative stress was investigated in the biofilm of *C. albicans.* For the analysis of ROS production with dihydrorhodamine 123 (DHR 123), the concentration of the peptide that showed pro-apoptotic activity was selected. Increased amount of intracellular ROS oxidizes non-fluorescent DHR to cationic rhodamine, which exhibits green fluorescence. Both peptides, ΔM3 (50 μM) and ΔM4 (6.2 μM) increased ROS production by approximately 38% compared to the untreated fungal biofilm (Figure 7B). At the same time, the expression of selected genes encoding antioxidant enzymes was tested at the peptide concentration inducing proapoptotic responses. The ΔM3 induced higher gene expression of *GPX3* and *SOD5* showing a 2-fold and 80-fold increase in mRNA level, respectively. The biofilm stimulation with ΔM4 peptide caused an increase in *GPX3* and *SOD5* mRNA level by 3.3- and 12.2-fold, respectively (Figure 7C). Additionally, a slight decrease in *CAT1* expression was observed when mature biofilms were treated with both peptides.

**Figure 8:**
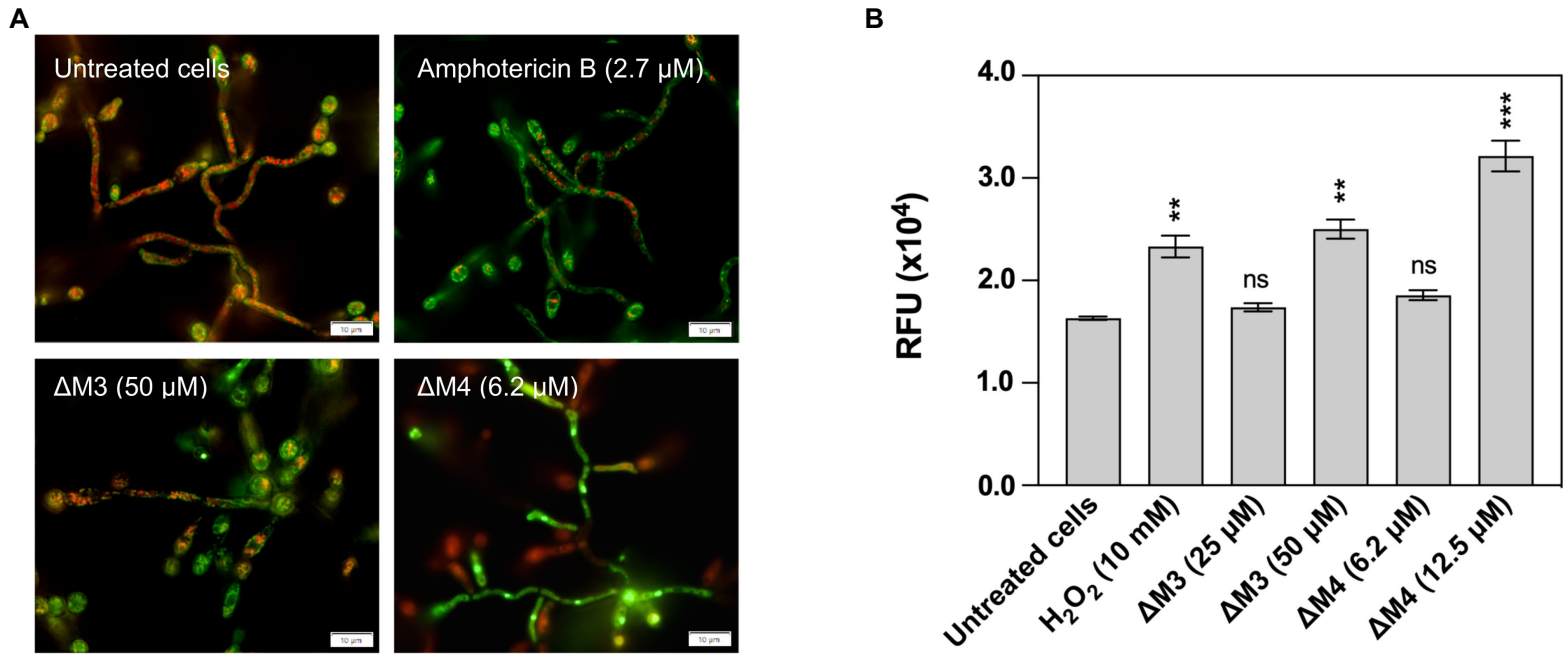
Induction of mitochondria activation and increased intracellular Ca^2+^ concentration by ΔM3 and ΔM4. **(A)** The changes in mitochondrial activity after treatment with peptides were detected in 24-h biofilms of *C. albicans.* Cells were incubated with 50 μM ΔM3, 6.2 μM ΔM4 or 2.7 μM amphotericin B for 3 h. Changes in mitochondrial activity and the entire mitochondrial pool was determined with 0.5 μM Mitotracker Orange and 0.5 μM Mitotracker Green, respectively. Representative images of microscope analysis performed with the Olympus IX73 microscope for each sample are presented. **(B)** To detect intracellular Ca^2+^, 24-h biofilms of *C. albicans* were incubated with ΔM3 (25 μM and 50 μM), ΔM4 (6.2 μM and 12.5 μM) or H_2_O_2_ (10 mM, positive control) for 3 h. Next the Fura-2 Calcium Flux Assay Kit was performed according to the manufacturer’s instructions. The bars represent the values of mean ± SD (*n* = 3), RFU – relative fluorescence unit. Statistical analysis was performed with one-way ANOVA with Dunnett’s multiple comparisons *post-hoc* test. **p < 0.01, ***p < 0.005.

To further characterize the action of the peptides on cell mitochondria, their functionality and dynamics were evaluated. Active mitochondria showing normal membrane potential were visualized with MitoTracker Orange, while the entire mitochondrial pool was determined by MitoTracker Green staining (Figure 8A). Representative photographs show a partial loss of mitochondrial activity after incubation with peptides and amphotericin B, as a decrease in MitoTracker Orange fluorescence was noted. In addition, the distribution of active mitochondria was altered in treated cells. Since mitochondrial function depends on intracellular Ca^2+^ concentration, the cytosolic concentration of these ions in peptide-treated *C. albicans* biofilm was also investigated. Intracellular Ca^2+^ mobilization in live cells was determined with a ratiometric calcium assay, in which the fluorescent indicator Fura-2 changes spectral properties when calcium ions are bound. Higher peptide concentrations (50 μM ΔM3 and 12.5 μM ΔM4) resulted in increased cytosolic Ca^2+^ concentration, while no effect was observed at lower peptide concentrations. The ΔM4 peptide more effectively stimulated cells to Ca^2+^ production (by approximately 100% compared to untreated cells), while ΔM3 showed an increase in Ca^2+^ concentration of approximately 70% (Figure 8B). In a positive control, cells were treated with 10 mM H_2_O_2_ and demonstrated an increase in Ca^2+^ concentration of up to 50%. These findings indicate that, at least in part, the antifungal activity of the peptides might be related to their pro-apoptotic properties.

### 3.5. The antifungal activity of ΔM3 and ΔM4 peptides was enhanced in combination with conventional antifungal drugs

Increased drug resistance of *Candida* spp. to conventional antifungal compounds, together with their toxicity, limits the effectiveness of candidiasis treatment (Tsui et al., 2016). In the fight against fungal infections, additional substances are often introduced in combination with conventional drugs to enhance the effect of treatment and to use lower concentrations of these, often toxic, drugs. This study investigated the effect of combined treatment of *C. albicans* with ΔM3 and ΔM4 peptides and conventional drugs, according to the modified checkerboard microdilution method in which *C. albicans* cells were seeded into microplate wells in RPMI with or without peptides, drugs or their combination over a wide range of two-fold serial dilutions of both agents, and then incubated for 24 h. To determine the inhibitory concentrations (IC) the XTT metabolic activity assay was used. The capacity for drug/peptide interaction with *C. albicans* was measured by calculating the fractional inhibitory concentration index (FICI), which was defined in the Materials and Methods section. A FICI value of less than or equal to 0.5 indicates that there is a drug and peptide synergism; a value between 0.5 and 1.0 indicates an additive effect of both substances, while a value between 1.0 and 2.0 indicates that the combination of the two substances is indifferent to cell effects. The FICI values calculated for the combination of ΔM3 and ΔM4 with amphotericin B, caspofungin, or fluconazole are presented in Table 3. Synergy against *C. albicans* was observed between caspofungin and each peptide. The FICI achieved a value of 0.062 and 0.370 for ΔM3 and ΔM4, respectively. In the case of the caspofungin/ΔM3 combination, the minimal inhibitory concentration that inhibits fungal metabolism by 90% was 0.003 μM and 1.6 μM for caspofungin and peptide, respectively. IC_90_ values for the caspofungin/ΔM4 combination were 0.012 μM and 0.8 μM for caspofungin and peptide, respectively. Due to the fact that the IC_90_ for these substances is 0.1 μM for caspofungin and 50 μM or 3.1 μM for ΔM3 and ΔM4, respectively (Table 2), it can be concluded that the treatment of cells with this drug in combination with the peptide strongly lowered the IC_90_. An additive effect was observed for the combination of peptides with amphotericin B, showing FICI values of 0.508 and 0.625 for ΔM3 and ΔM4, respectively. As with the caspofungin/peptide combination, in this case, the IC_90_ for the drug combination is reduced compared to the effect achieved by administering these substances alone, reaching values of 0.8 μM for amphotericin B and 0.4 μM for peptides. In turn, the effect of combining peptides with fluconazole turned out to be indifferent. The calculated FICI values were equal to 1.25 for both peptides. In fact, the IC_90_ values for the peptides were the same as the values obtained when the peptides were used alone. However, IC_90_ for fluconazole was four-fold lower when administered in combination with each peptide than when administered alone. Inhibition curves for each drug/peptide combination are presented in Supplementary Figure S2. They indicate that both peptides are useful for reducing the dose of conventional drugs such as caspofungin and amphotericin B in inhibiting the growth of *C. albicans.*

**Table 3:** Combined antifungal activity of ΔM3 and ΔM4 with conventional antifungals.

Compound a	Compound b	FICa	FICb	FICI	Action
Amphotericin B	ΔM3	0.5 (0.8 μM)	0.008 (0.4 μM)	0.508	Additive
	ΔM4	0.5 (0.8 μM)	0.125 (0.4 μM)	0.625	Additive
Caspofungin	ΔM3	0.03 (0.003 μM)	0.032 (1.6 μM)	0.062	Synergism
	ΔM4	0.12 (0.012 μM)	0.25 (0.8 μM)	0.37	Synergism
Fluconazole	ΔM3	0.25 (0.2 μM)	1.0 (50 μM)	1.25	Indifferent
	ΔM4	0.25 (0.2 μM)	1.0 (3.1 μM)	1.25	Indifferent

The combinatory effect of peptides and antifungal drugs was determined using the modified checkerboard assay. *C. albicans* cells (3 × 10^3^/ml) were treated with ΔM3, ΔM4, amphotericin B, caspofungin, fluconazole alone or with mixtures in a wide concentration range for 24 h at 37°C in RPMI 1640 medium. The minimal concentration that inhibits the metabolic activity of fungi by 90% (IC_90_) for each compound and each combination of compounds was determined using the XTT reduction assay in at least three biological replicates. IC_90_ determined for self-acting peptides and drugs: 50 μM for ΔM3, 3.1 μM for ΔM4, 1.6 μM for amphotericin B, 0.8 μM for fluconazole, and 0.1 μM for caspofungin. The IC_90_ values for the compounds acting in the combination are given in brackets. FICI values: FICI ≤0.5 = synergism, 0.5< FICI ≤1.0 = additive, 1.0< FICI ≤2.0 = indifferent, FICI >2.0 = antagonism.

### 3.6. The viability of human dermal fibroblasts varies depending on the peptide used

**Figure 9:**
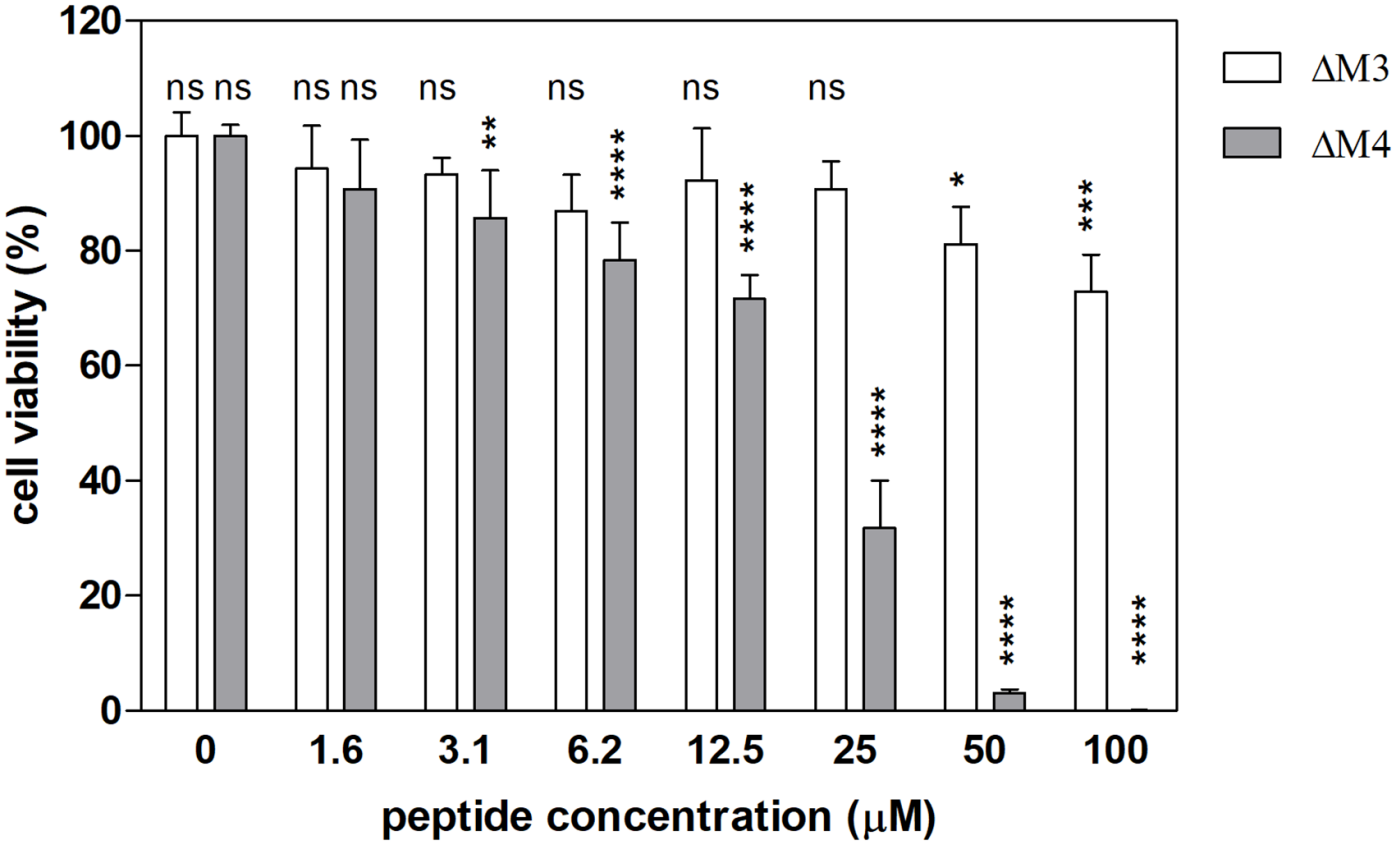
Viability of human dermal fibroblasts (HDF) after treatment with ΔM3 and ΔM4 peptides. The cytotoxicity of the peptides was assessed with Alamar blue test in primary HDF. Cells, seeded on microplate (1 × 10^5^), were treated with peptides at different concentrations for 24 h in culture HDF basal medium. After medium removal, 100 μl of Alamar blue reagent were added. Fluorescence was measured at λ_ex_ = 560 nm, λ_em_ = 590 nm with Synergy H1 microplate reader after 1 h of incubation. The cell viability is presented as the percentage of the untreated cells, the number of which was assumed as 100%. The samples were performed in triplicate. The bars present the mean ± SD (*n* = 3). Statistical analysis was performed with two-way ANOVA with Tukey’s multiple comparison test. Selected statistical significances were presented vs. untreated cells. *p < 0.05, **p < 0.01, ***p < 0.005, ****p < 0.001.

The cytotoxicity of the tested peptides was determined in primary human dermal fibroblasts (HDF) with the Alamar blue assay. Living cells convert the resazurin indicator to highly fluorescent resorufin. The effects of the two peptides on fibroblast viability differed (Figure 9). ΔM3 peptide caused only a slight decrease in cell viability, achieving the major effect at the concentration of 100 μM (by 24%). Differently, the second peptide, ΔM4 was more aggressive toward fibroblasts, causing a 25% decrease in cell viability at the concentration of 12.5 μM. Higher concentrations of this peptide resulted in a strong reduction in cell viability, leading to a complete cell killing at 100 μM.

## 4. Discussion

The incidence of fungal infections and their mortality rates have increased significantly in recent years, particularly for candidiases which are most common (Pfaller et al., 2020). *C. albicans* still remains the predominant etiological agent of candidiasis; however, non-*albicans Candida* species, including *C. parapsilosis*, *C. tropicalis*, and *C. glabrata*, are emerging as major opportunistic fungal pathogens, causing increased rate of hospital-acquired infections, with high risk for immunosuppressed patients undergoing surgical procedures or anticancer therapy (Pfaller et al., 2019). These pathogens also often present a reduced susceptibility to commonly used antifungal drugs, due to the increase in frequency of multidrug resistant strains or the formation of biofilms that protect them from conventional therapies (Mayer et al., 2013). Therefore, successful treatment of *Candida* infections that are often associated with biofilm formation requires novel and more effective antifungal strategies. The selection of efficient antimicrobial peptides possesses some advantages over conventional antifungal agents, the effectivity of which can be reduced by their toxicity toward human host cells. Cecropins have recently become an important source of AMPs, which, thanks to their specific structure and interactions with the lipid membranes of various cells, present antimicrobial and anticancer effects (Hollmann et al., 2018). It has been found that AMPs may use two or more mechanisms to penetrate membranes that depend on the polar and non-polar distribution of amino acids; however, other characteristics such as net charge, amphipathicity, hydrophobicity, percentage of α-helix structure may be important for interaction with the cell wall (Madani et al., 2011). Recent reports have demonstrated the effect of cecropin-derived amphipathic positive peptides on antifungal activity against *C. albicans* (Peng et al., 2022; Sun et al., 2022). These studies, however, focused on the antifungal activity of peptides that differ in amino acid sequence, charge, and hydrophobicity from the peptides tested in this study.

To test antifungal activity on different forms of *Candida* cells, we chose two cecropin D-derived cationic peptides, ΔM3 and ΔM4, which are both rich in Lys and Arg residues, while ΔM4 peptide was additionally enriched with three Trp residues and one Tyr residue, thus presenting more hydrophobic properties compared to ΔM3. Differences in amino acid composition resulted in different predisposition of peptides to the formation of α-helical structures in the presence of agents, simulating contact with the microbial cell membrane, as determined by the CD study. Furthermore, the presence of salt in the environment can promote this property with respect to the peptide ΔM4, while such effect was not observed for ΔM3. These structural properties turn directly into the effectiveness of these peptides in contact with yeast cells, especially those that occur in the form of blastospores. In fact, we observed different patterns of the fungal cell surface damage for both investigated peptides by SEM. In accordance with our results, similar observations were reported, showing that incorporation of hydrophobic amino acid residues in AMPs resulted in reduced MIC_90_ values (Pandit et al., 2020; Ramamourthy et al., 2020). Furthermore, the presence of hydrophobic residues in the transmembrane fragment of antimicrobial peptides was believed to lead to the formation of ion channels within the cell membrane (Buda De Cesare et al., 2020). The amino acid composition also influenced the differences in fungicidal activity of the peptides studied, depending on the presence of ions in the environment. The ΔM3 peptide, which has a charge of +8, appeared to be more susceptible to the salt effect, regardless of the ability to form helical structures, since no activity against *C. albicans* was observed after treatment of fungal cells with a peptide dissolved in PBS, compared to the strong activity detected when HEPES buffer was used. Such a negative impact of salt on the fungicidal activity of short cationic peptides against *C. albicans* has previously been demonstrated (Do Nascimento Dias et al., 2020; Ghosh et al., 2021). It may be the result of compensation for the peptide charge or of affecting the local peptide structure by electrostatic interactions of ions with amino acid functional groups (Baldauf et al., 2013). Contrary, ΔM4 was able to kill *C. albicans* even in PBS but with a lower efficiency compared to the results obtained in HEPES buffer. Such differences can be explained by different mechanisms and/or kinetics of both peptide action, where membrane compromising activity is accompanied by more complex and diverse mechanisms including DNA damage, inhibition of protein synthesis or metabolic pathway, induction of apoptosis, and oxidative stress (Nguyen et al., 2011; Wang et al., 2015; Li et al., 2016). For example, in the case of the application of the tryptophan-rich PuroA peptide, its rapid translocation inside *C. albicans* cells and its binding to intercellular targets was proposed as a mechanism of yeast inactivation, before subsequent perturbation of the cell membrane (Shagaghi et al., 2017). Another component of the incubation solution that radically inhibited the antifungal activity of the tested peptides was human plasma. Similar observations have also been reported for several AMPs, including LL-37. One study pointed to the possibility of forming complexes between peptides and plasma proteins such as albumin/α1-antitrypsin complex or apolipoprotein AI, resulting in the inactivation of AMPs (Tang et al., 2021).

Preliminary studies on other *Candida* species have been performed, indicating that the susceptibility of *C. albicans, C. tropicalis,* and *C. parapsilosis* cells to both peptides was comparable, while *C. glabrata* cells were killed at higher concentrations of peptides. This was expected as *C. glabrata* is known to present a low susceptibility to azoles (Arendrup et al., 2013) and a significant resistance to amphotericin B (Pappas et al., 2016), which was proposed to be associated with the presence of fungal cell wall transporters and their increased activity in the export of antifungal agents (Cavalheiro et al., 2021). Limited susceptibility of *C. glabrata* to AMPs was also observed in the treatment with histatin 5, magainin 2, cathelicidins, and defensins (Helmerhorst et al., 2005; Del Gaudio et al., 2013).

In the presented study, the observed influence of peptides on fungal cell adhesion and filamentation was also significant, as the regulation of these processes may prevent biofilm formation and fungal cell resistance to treatment. A noticeable inhibition of fungal cell filamentation was observed at a concentration of the peptides lower than fungicidal, and it was manifested by the formation of shorter hyphae and decreased expression of genes encoding proteins characteristic for the filamentous form of *C. albicans* cells (Hwp1, Hyr1, and Als3). A recent study demonstrated a similar effect on *C. albicans* by the cecropin-derived peptide C18 (Sun et al., 2022). It should be noted that the arresting of fungal cells in the form of blastospores may facilitate their elimination, even at lower peptide concentration. Such a strategy has been proposed for many antimicrobial peptides (Ogasawara et al., 2007; Akerey et al., 2009; Theberge et al., 2013; Do Nascimento Dias et al., 2020). The development of hyphae by *C. albicans* cells is a hallmark of the initial steps in biofilm formation (Tsui et al., 2016). Then the consequence of the observed inhibition of hypha production by the peptides was the disruption of biofilm development, which was identified at the same peptide concentration range.

The diagnosis of a yeast infection and the necessity for its treatment often come at the stage of a biofilm formed. Therefore, we also tested the efficacy of both peptides against a mature biofilm, with a developed matrix, which hinders the penetration of various antifungal drugs, forcing an increase in the dose of the drug, which in turn could be toxic to the cells of the treated host (Taff et al., 2013; Cavalheiro and Teixeira, 2018). For ΔM4 peptide, as in the case of many other AMPs (Kodedová and Sychrová, 2017; Do Nascimento Dias et al., 2020), we observed a noticeable decrease in the metabolic activity of mature biofilms at a peptide concentration approximately 15-fold or 8-fold higher compared to its effectiveness against yeast-like or biofilm-initiating cells. However, these concentrations of ΔM4 were close to its cytotoxic properties toward host cells, where HDF viability decreased gradually with increasing ΔM4 concentration, and was eliminated at the ΔM4 concentration of 100 μM. In turn, the ΔM3 peptide showed a lower toxicity in relation to HDF cells; at a concentration of 100 μM fibroblast viability was decreased only by 25%. However, at this concentration, the peptide lost the ability to eradicate the mature biofilm. Regardless of the effect of the peptides on host cells, it can be concluded that at least in the case of ΔM4, all morphological forms of *C. albicans* can be destroyed. The second peptide, ΔM3, worked better on blastospores and, at high concentrations, can significantly reduce pre-formed and mature biofilms.

Although the effects of AMPs on microorganisms are mainly attributed to their interaction with the cell membrane, other mechanisms have also been identified, including inhibition of cell wall biosynthesis or interaction with DNA (Buda De Cesare et al., 2020). However, certain peptides, such as salivary histatin 5 (Helmerhorst et al., 2001) or the venom wasp protonectin (Wang et al., 2015) involved also ROS production in their antifungal mechanism and induced oxidative stress in yeasts through uncontrolled accumulation of intracellular ROS, which led to cell apoptosis or necrosis (Kim et al., 2020; Seyedjavadi et al., 2020; Zhang et al., 2021). For ΔM3 and ΔM4 peptides, increased cell permeability was also accompanied by increased ROS production within the *C. albicans* biofilm, suggesting a more complex antifungal mechanism of peptide action. However, *C. albicans* possesses adaptive responses that allow ROS detoxification, including activation of superoxide dismutase, catalase, and other enzymes involved in the production of ROS scavengers (Jamieson et al., 1996; Dantas et al., 2015). Activation of these reactions was also observed after treatment of the *C. albicans* biofilm with ΔM4 peptide, where increased expression of the *GPX*3 and *SOD5* genes was detected. However, the expression of *CAT1* was reduced, suggesting that ROS detoxification upon ΔM4 treatment may not be completed. The explanation for such a result was indicated by Kaloriti et al. (2014), who showed that simultaneous exposure of *C. albicans* cells to oxidative and cationic stress may be more effective in yeast killing than oxidative stress alone (Kaloriti et al., 2014). These authors also demonstrated that cationic ions can inhibit catalase, leading to ROS accumulation and inhibition of the Cap1 signaling molecule, which is critical for the response to oxidative stress of *C. albicans* cells.

Mitochondria are considered the main source of ROS, but uncontrolled ROS production also makes mitochondria a target, influencing mitochondrial membrane functionality. As a result, changes in membrane potential, increased mitochondrial permeability, and finally mitochondrial apoptosis are observed (Choi and Lee, 2015). In this study, mitochondrial functionality was investigated with fluorescence dyes, which identify active mitochondria with normal membrane potential versus the total number of mitochondria in *C. albicans* cells that form a biofilm, treated with both peptides. In each case, a significant loss of active mitochondria was observed, confirming that peptides contribute to biofilm disruption. These organelles develop different repair mechanisms, including mitochondrial fission-fusion processes, which are important for their proper functioning (Youle and van der Bliek, 2012). A previous study showed that *C. albicans* can lose its virulence due to the loss of the ability of mitochondria to fuse (Liang et al., 2018). It is possible that biofilm treatment with the peptides can induce such processes, but to confirm this observation, more research is needed.

The fact that the tested peptides increased ROS production and disrupted mitochondrial functionality was confirmed by the detection of increased cytosolic Ca^2+^ concentration after peptide treatment of *C. albicans* cells. On the other hand, the accumulation of Ca^2+^ violates its homeostasis and inhibits mitochondrial respiration, leading to its dysfunction (Rossi et al., 2019). Several studies have demonstrated that high cytosolic Ca^2+^ concentration causes an increased influx of these ions into mitochondria, resulting in increased permeability and depolarization of the mitochondrial membrane, and enhanced ROS production by *C. albicans* (Lee and Lee, 2018; Niu et al., 2020; Kwun et al., 2021). Furthermore, it has been shown that deregulated calcium ion homeostasis can induce *C. albicans* apoptosis (Kwun et al., 2021). Our study demonstrated increased metacaspase activity in the *C. albicans* biofilm treated with both peptides, which confirms the development of apoptotic processes in the treated biofilm. Previously, the apoptotic effects of various peptides have been demonstrated for *Candida* yeast cells (Lee and Lee, 2014; Li et al., 2019; Chen and Lan, 2020; Kim et al., 2020). In summary, taking into account increased ROS production and Ca^2+^ concentration, enhanced mitochondrial dysfunction, and increased metacaspase activity, we can propose a partial pro-apoptotic mechanism in the fungicidal activity of the studied peptides.

Strategies to reduce peptide toxicity include peptide further modification or encapsulation (van Gent et al., 2021). Combining them with conventional antifungal drugs is also a good approach, especially because the use of drug combinations can not only improve the efficiency of both antifungal agents, often with lowering their active concentration range and reducing their cytotoxic effect, but also can decrease the development of the resistance. Therefore, we evaluated the effects of each of the peptides along with amphotericin B, caspofungin and fluconazole that resulted in three different types of effects. In the first type of interaction, synergism was observed only for the combination of peptides with caspofungin, especially strong in the case of ΔM3, for which a 30-fold reduction in IC_90_ was identified (Table 3). Similar observations have been reported for the combination of caspofungin with many other AMPs (Vriens et al., 2015; Kovács et al., 2019; Shen et al., 2021). Caspofungin inhibits β-1,3-glucan synthase, located in the cell membrane of *Candida* cells, responsible for an essential component of the fungal cell wall. The reduced content of glucan in the fungal cell wall may facilitate its better penetration by the peptide. The second type of action, additivity, was determined for the combination of the peptides with amphotericin B. For both peptides, a two-fold reduction in IC_90_ was observed in cooperation with amphotericin B. However, it was found that the concentration of peptides needed to inhibit the formation of *C. albicans* biofilms to 10% was 125- and 8-fold lower for amphotericin B combination with ΔM3 and ΔM4, respectively, compared to peptides acting alone. Although the interaction between these compounds has not been classified as synergistic, it is relatively strong and after the verification by different types of tests or *in vivo* studies (Vitale et al., 2005) it might change character, as it was observed previously in the case of caspofungin/amphotericin B combination used against *C. parapsilosis* (Barchiesi et al., 2007). When peptides were combined with fluconazole, no changes in antifungal properties were observed. Although the determined FICI of 1.25 suggests in both cases an independent effect, where both peptides clearly negatively affect the antifungal activity of fluconazole, as it was detected even at the lowest peptide concentration tested (0.4 μM). However, the negative effect of fluconazole on the antifungal activity of both peptides was not detected (Supplementary Figure S2). Trapping fluconazole by peptides and consequently the drug’s failure to reach its target site without affecting its own antimicrobial activity is one of the possible reasons for the observation of such a phenomenon. It is also possible that the peptides, like some other AMPs (Ma et al., 2020), may increase the expression of Erg11, an essential enzyme in the ergosterol biosynthesis pathway, which is inhibited by fluconazole. Moreover, it is also not excluded that the peptides can increase expression and/or activity of *C. albicans* efflux pumps and thus increase the fungus resistance to fluconazole but it needs further research.

**Figure 10:**
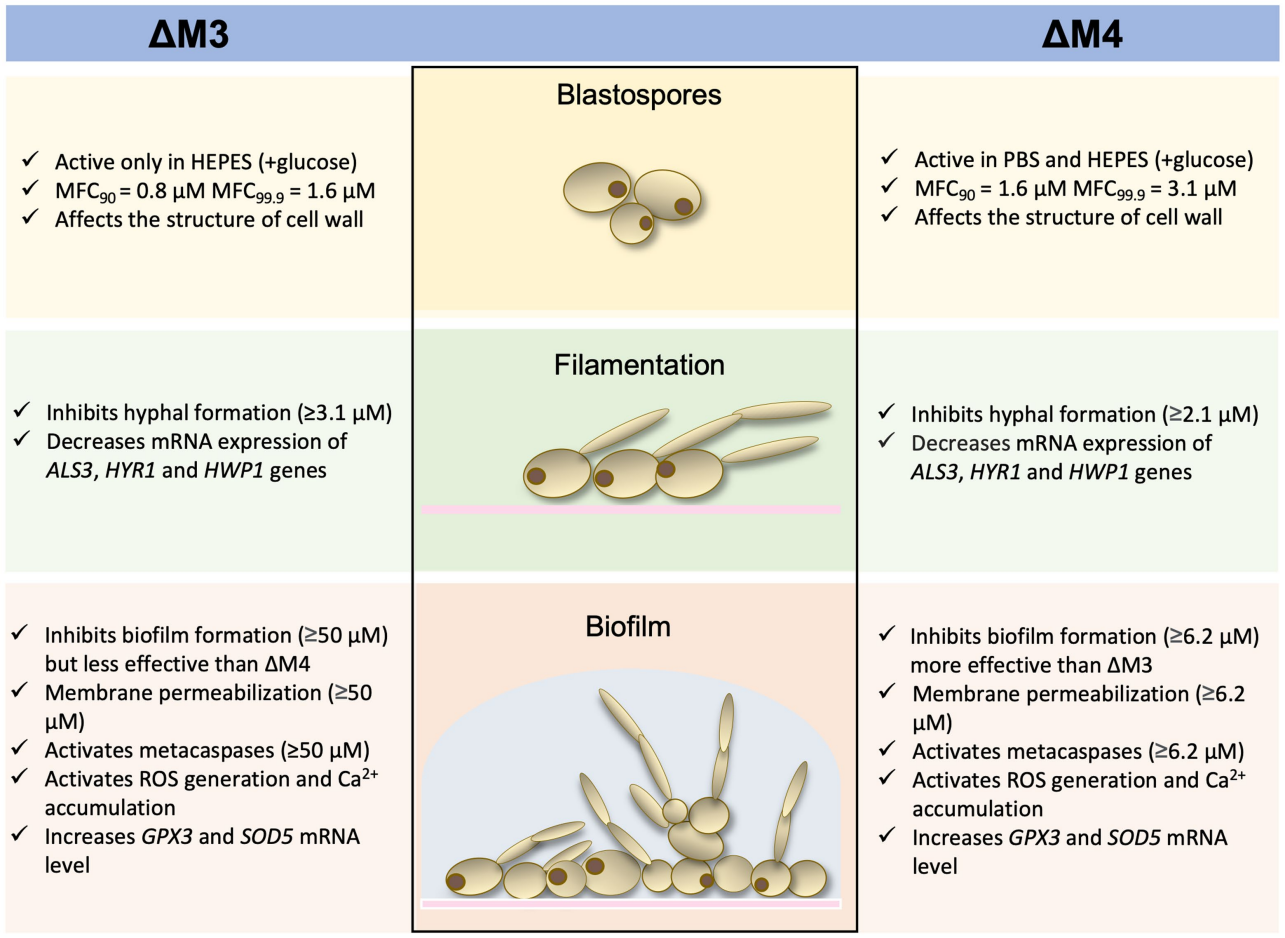
Diagram presenting a summary of the results.

In conclusion, cecropin D-derived synthetic cationic peptides presented strong antifungal activity against *Candida* cells, regardless of the morphological form of the fungi. A scheme that demonstrates the most relevant results is presented in Figure 10. As expected, yeast cells of *C. albicans* were more susceptible to the action of peptides compared to the fungal biofilm. The mode of action of peptides is most likely complex, involving both destabilization of the cell wall and additional intracellular mechanisms such as increased oxidative stress, which contributes to cell death by apoptosis. Moreover, the synergism of tested peptides with caspofungin was demonstrated. That offers an opportunity for a new approach to combating *Candida* infections by constraining their virulence and dealing with drug resistance.

## Data availability statement

The original contributions presented in the study are included in the article/Supplementary material, further inquiries can be directed to the corresponding author.

## Author contributions

IG-L: methodology, investigation, formal analysis, conceptualization, writing-original draft, and writing-review and editing. GB: methodology, investigation, formal analysis, funding acquisition, writing-original draft, and writing-review and editing. MJ and AG: methodology, investigation, and formal analysis. JK-K: investigation and writing-review and editing. MM-M and EP: resources and writing-review and editing. JD: methodology and investigation. AK: writing-review and editing and funding acquisition. MR-K: conceptualization, supervision, funding acquisition, writing-original draft, and writing-review and editing. All authors contributed to the article and approved the submitted version.

## Funding

This work was supported by the National Science Centre of Poland (grant no. 2019/33/B/NZ6/02284 awarded to MR-K) and the Jagiellonian University in Krakow and the Statutory Funds of the Faculty of Biochemistry, Biophysics and Biotechnology (project No. N19/MNS/000035 awarded to GB).

## Conflict of interest

The authors declare that the research was conducted in the absence of any commercial or financial relationships that could be construed as a potential conflict of interest.

## Publisher’s note

All claims expressed in this article are solely those of the authors and do not necessarily represent those of their affiliated organizations, or those of the publisher, the editors and the reviewers. Any product that may be evaluated in this article, or claim that may be made by its manufacturer, is not guaranteed or endorsed by the publisher.

## Supplementary material

The Supplementary material for this article can be found online at: https://www.frontiersin.org/articles/10.3389/fmicb.2022.1045984/full#supplementary-material
