# Supplementary material for: Cecropin D-derived synthetic peptides in the fight against Candida albicans cell filamentation and biofilm formation
Source: Front Microbiol. 2023 Jan 13;13:1045984. doi: 10.3389/fmicb.2022.1045984 (PMC9880178; doi:10.3389/fmicb.2022.1045984)
Supplement: Supplementary file 1 [file Data_Sheet_1.PDF]

Cecropin D-derived synthetic peptides in the fight against *Candida albicans* cell filamentation and biofilm formation

Ibeth Guevara-Lora[1]†, Grazyna Bras[2]†, Magdalena Juszczak[2], Justyna Karkowska-Kuleta[2], Andrzej Gorecki[3] , Marcela Manrique-Moreno[4], Jakub Dymek[5], Elzbieta Pyza[5], Andrzej Kozik[1], Maria Rapala-Kozik[2*]

[1] Department of Analytical Biochemistry, Faculty of Biochemistry, Biophysics and Biotechnology, Jagiellonian University, Krakow, Poland

[2] Department of Comparative Biochemistry and Bioanalytics, Faculty of Biochemistry, Biophysics and Biotechnology, Jagiellonian University, Krakow, Poland

[3] Department of Physical Biochemistry, Faculty of Biochemistry, Biophysics and Biotechnology, Jagiellonian University, Krakow, Poland

[4] Chemistry Institute, Faculty of Exact and Natural Sciences, University of Antioquia, Medellin, Colombia

[5] Department of Cell Biology and Imaging, Institute of Zoology and Biomedical Research, Jagiellonian University, Kraków, Poland

*** Correspondence:**
Maria Rapala-Kozik
maria.rapala-kozik@uj.edu.pl

† These authors share first authorship

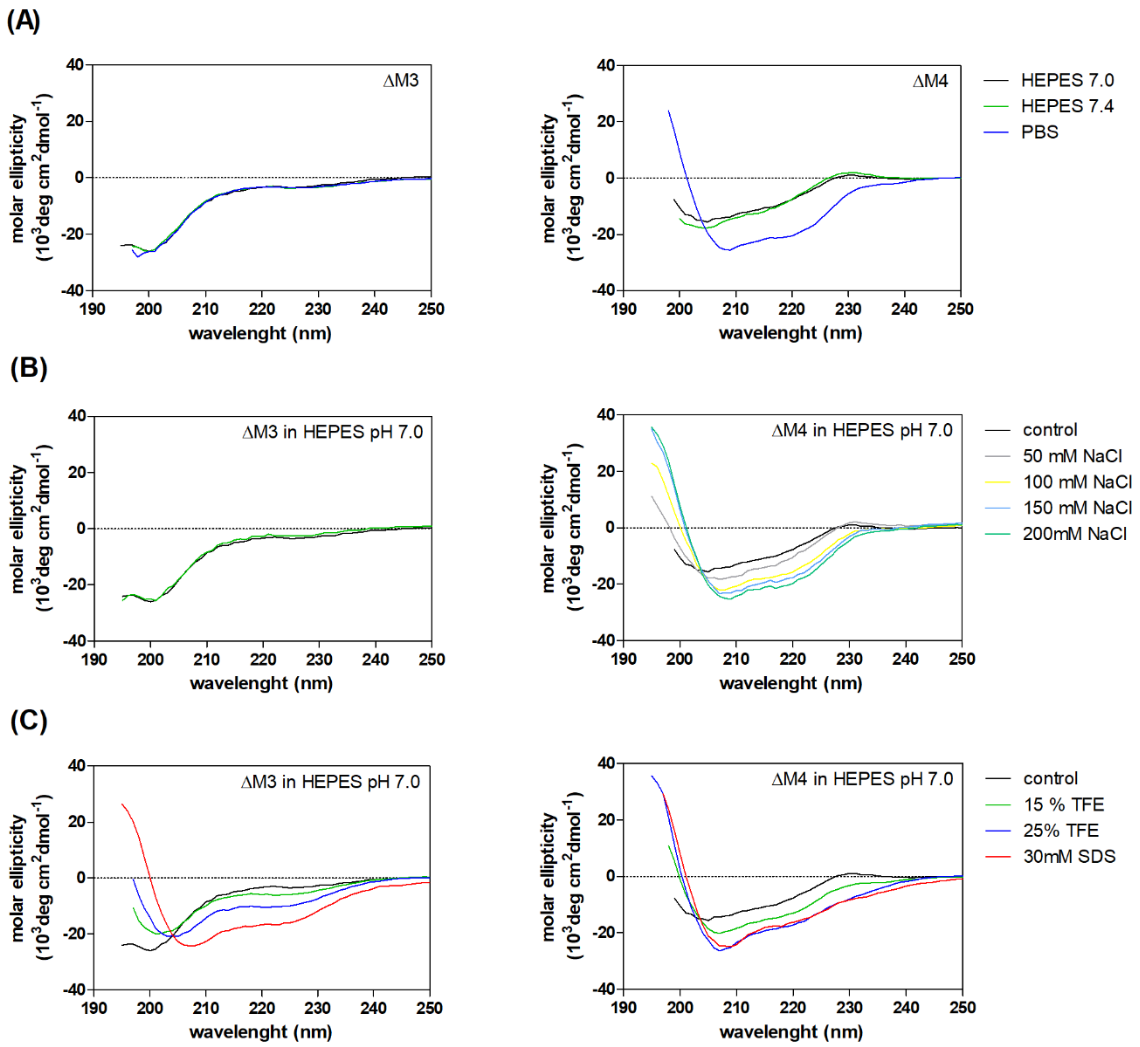

Figure S1.

Circular dichroism (CD) spectra of ΔM3 and ΔM4. (A) The peptide spectra recorded in 10 mM HEPES pH 7.0, 10 mM HEPES pH 7.4 and PBS. (B) The peptide spectra recorded in 10 mM HEPES pH 7.0 without (control) or with NaCl in the concentration range of 50 mM to 200 mM, at 30°C. (C) Selected CD spectra of peptides measured in the presence of trifluoroethanol (TFE) and sodium dodecyl sulphate (SDS). The peptides diluted in 10 mM HEPES pH 7.0 (control) and in the same buffer supplemented with 15% and 25% TFE or 30 mM SDS were prepared. All spectra were recorded in the range of 195–250 nm with 1 nm data pitch, 20 nm/min scanning speed, 4 s integrating time, 2 nm bandwidth, in quartz cuvettes of 0.01 cm path-length at 30°C and averaged over five acquisitions. In all cases, the peptide concentration was 1 mg/ml. All spectra were corrected for the effect from the buffer, and all measurements were converted to molar residual ellipticity units.

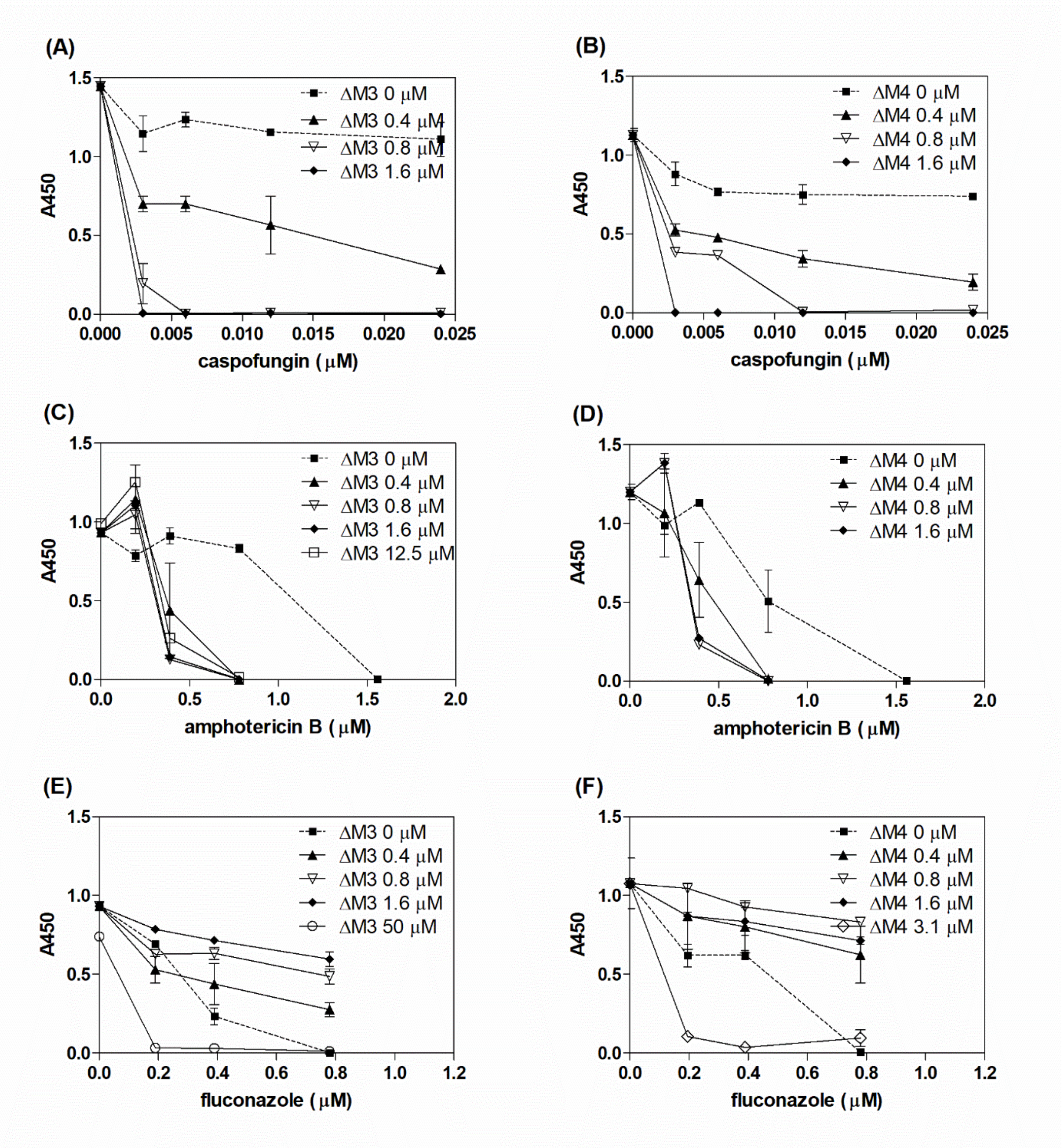

Figure S2.

Combined antifungal activity of ΔM3 and ΔM4 with conventional drugs. The combinatory effect of peptides and antifungal drugs was determined using the checkerboard assay with metabolic activity detection. *C. albicans* ($3x10^3$ cells/ml) was treated with ΔM3 (A, C, E), ΔM4 (B, D, F), caspofungin (A, B), amphotericin B (C, D), fluconazole (E, F) alone or with a combination of peptide and drug in a wide range of concentrations for 24 hours at 37°C in RPMI medium, in 96-well microplate. After incubation, the metabolic activity of *C. albicans* biofilm cells was determined using an XTT reduction assay (A450 – absorbance of formazan product, which is proportional to the metabolic activity of fungi). Representative results of three independent experiments are presented as mean ± SD (n=2).
